# Timing of Exposure to Parental Depression From Pregnancy to Young Adulthood and Mental Health in Adult Offspring

**DOI:** 10.1001/jamanetworkopen.2026.4892

**Published:** 2026-04-10

**Authors:** Adelaide Feibel, Hung Pham, Vivette Glover, Thomas G. O’Connor, Kieran J. O’Donnell

**Affiliations:** 1 Yale Child Study Center, Yale School of Medicine, New Haven, Connecticut; 2 Department of Obstetrics, Gynecology and Reproductive Sciences, Yale School of Medicine, New Haven, Connecticut; 3 Institute of Reproductive and Developmental Biology, Imperial College London, United Kingdom; 4 Department of Psychiatry, University of Rochester School of Medicine and Dentistry, Rochester, New York; 5 Wynne Center for Family Research, University of Rochester School of Medicine and Dentistry, Rochester, New York; 6 Department of Neuroscience, University of Rochester School of Medicine and Dentistry, Rochester, New York; 7 Department of Obstetrics and Gynecology, University of Rochester School of Medicine and Dentistry, Rochester, New York; 8 Department of Psychology, Yale University, New Haven, Connecticut

## Abstract

**Importance:**

Exposure to parental depression is a risk factor for offspring mental illness.

**Objective:**

To examine the association between the timing of exposure to parental depression, from pregnancy to young adulthood, and adult offspring mental health.

**Design, Setting, and Participants:**

This prospective longitudinal cohort study of adult offspring aged 22 to 27 years in the Avon Longitudinal Study of Parents and Children, a British birth cohort, was conducted from September 1990 to July 2020. Data were analyzed from March 2024 to January 2026.

**Exposures:**

Parental depressive symptoms were assessed repeatedly using the Edinburgh Postnatal Depression Scale (score range, 0 to 30, with higher scores indicating more severe depressive symptoms) beginning in pregnancy through offspring age of 21 years.

**Main Outcomes and Measures:**

The main outcomes were offspring symptoms of depression at age 27 years, anxiety at age 25 years, psychotic disorders at age 24 years, and alcohol use disorder (AUD) at age 22 years. Covariates included socioeconomic status and maternal-offspring polygenic risk for multiple psychiatric disorders.

**Results:**

A total of 5329 adult offspring (3276 females [61.5%]) provided at least 1 outcome measure, which included 3795 participants providing symptoms of depression (mean [SD] age, 27.8 [0.5] years), 3505 participants providing symptoms of anxiety (mean [SD] age, 25.3 [0.6] years), 3342 participants with assessments for psychotic disorders (mean [SD] age, 24.5 [0.8] years), and 3392 participants reporting on symptoms of AUD (mean [SD] age, 22.9 [0.5] years). Cumulative exposure to parental depression across all time points was associated with increased odds of offspring depression (maternal: AOR, 2.36 [95% CI, 1.91-2.92]; paternal: AOR, 2.13 [95% CI, 1.60-2.83]) and anxiety (maternal: AOR, 2.58 [95% CI, 2.06-3.23]; paternal: AOR, 1.98 [95% CI, 1.49-2.63]). Only maternal depression was associated with increased odds of psychosis symptoms (maternal: AOR, 1.90 [95% CI, 1.27-2.82]; paternal: AOR, 1.63 [95% CI, 0.95-2.80]). There were no statistically significant associations with AUD. Significant associations between maternal depression and adult offspring depression were observed from 32 weeks’ gestation (AOR, 1.08 [95% CI, 1.01-1.15]) to age 18 years (AOR, 1.08 [95% CI, 1.01-1.16]). Maternal depression from the 8-month postnatal period (AOR, 1.06 [95% CI, 1.01-1.11]) onward (aged 21 years: AOR, 1.13 [95% CI, 1.02-1.24]) was associated with offspring anxiety symptoms. Paternal depression was significantly associated with offspring depression from mid-childhood (AOR, 1.08 [95% CI, 1.01-1.15]) onward (aged 21 years: AOR, 1.22 [95% CI, 1.04-1.43]), with similar associations between paternal depression and offspring anxiety from mid-childhood (aged 5 years: AOR, 1.11 [95% CI, 1.03-1.18]) onward (aged 21 years: AOR, 1.22 [95% CI, 1.04-1.43]). Only maternal prenatal depression at 32 weeks’ gestation was associated with offspring psychotic symptoms (AOR, 1.20 [95% CI, 1.03-1.41]).

**Conclusions and Relevance:**

In this cohort study, analyses of 2 decades of data found distinct temporal associations between maternal and paternal depression and offspring psychiatric symptoms, and pregnancy was found to be a sensitive period in the association between maternal depression and offspring psychotic experiences. The findings suggest a substantial role of timing for specifying the association between parental depression and psychiatric outcomes in young adults and emphasize the need to support parental mental health from pregnancy onward.

## Key Points

**Question:**

Is there an association between the timing of exposure to parental depression, from pregnancy to age 21 years, and adult offspring mental health?

**Findings:**

In this cohort study of 5329 adult offspring who participated in the Avon Longitudinal Study of Parents and Children, distinct patterns of associations between the timing of exposure to maternal and paternal depression and adult offspring mental health symptoms were found. Maternal prenatal depressive symptoms were uniquely associated with offspring psychotic symptoms.

**Meaning:**

The findings of this study highlight the importance of supporting parental mental health in pregnancy and throughout child development.

## Teaser

This cohort study examines whether the timing of exposure to parental depression, from pregnancy through young adulthood, is associated with adult offspring mental health.

## Introduction

Exposure to maternal depression during pregnancy more than doubles the risk of adverse mental health outcomes in childhood, adolescence, and young adulthood.^1,2,3,4^ Daughters of mothers who experience prenatal depression are approximately 5 times more likely to report depression in adolescence and are 3 times more likely to be depressed in their own pregnancies.^3,5^ Such findings imply that pregnancy may be a sensitive exposure period (ie, a period during which exposures may have a particularly strong or lasting impact on offspring mental health).

Identifying sensitive periods of exposure to parental depression requires frequent assessment because of the dynamic change in symptom expression over time, particularly during the transition to parenthood.^6,7^ Prior studies seeking to demonstrate a sensitive period for exposure to maternal depression on offspring mental health focus from the postpartum period onward, but there is clear evidence that in utero exposure must also be considered,^8,9,10^ with more limited but notable findings highlighting the importance of paternal perinatal depression.^11,12^ Few studies that have incorporated prenatal assessments have examined mental health in early adulthood when the majority of psychiatric illnesses have manifested^13^ or have focused on 1 outcome.^5,14^ Additionally, given the familial nature of psychiatric disorders, the possible contribution of shared genetic risk factors must be considered when quantifying the association between parental depression and offspring mental illness.^2,15,16,17,18^

The Avon Longitudinal Study of Parents and Children (ALSPAC), a British birth cohort, provides repeated assessments of maternal and paternal depression over the first 2 decades of life, beginning in pregnancy, paired with maternal and offspring genetic data.^19,20^ In this study, we examined whether the developmental timing of parental depression exposure may be associated with multiple domains of mental health in adult offspring using distributed lag models (DLMs).^21^

## Methods

### Study Design

ALSPAC enrolled pregnant women participants from a geographical area formerly known as Avon County with due dates between April 1, 1991, and December 31, 1992.^20^ A total of 14 541 pregnancies were included in the original sample. Subsequent recruitment brought the sample size to 15 447 pregnancies with data available after 7 years.^19,22^ Partners were also invited to complete questionnaires. Data collection within ALSPAC began in September 1990 and is on-going. Ethical approval for the study was obtained from the ALSPAC Ethics and Law Committee and the Local Research Ethics Committees. Written informed consent for the use of all data collected was obtained from participants following the recommendations of the ALSPAC Ethics and Law Committee at the time. This study followed the Strengthening the Reporting of Observational Studies in Epidemiology (STROBE) reporting guideline for cohort studies.

### Participants

Adult offspring ALSPAC participants who completed mental health surveys or clinical interviews, with at least 1 measure of parental depression, and met study entry criteria: gestational age exceeding 32 weeks and birth weight greater than 1500 g. The last survey included in the current study was administered between November 2019 and July 2020.

### Measures

Parental depression was measured using the Edinburgh Postnatal Depression Scale (EPDS), a 10-item questionnaire (in which scores range from 0 to 30, with higher scores indicating more severe depressive symptoms), validated for use in pregnancy, postpartum, outside the perinatal period, and in fathers.^23,24,25^ When used as a screening tool, an EPDS score of 13 or more is commonly used as a threshold for depressive symptoms of clinical concern.^23^

Mothers completed EPDS questionnaires twice during pregnancy (at 18 weeks’ gestation and 32 weeks’ gestation) and when their children were approximately aged 8 weeks; aged 8, 21, and 33 months; and aged 5, 6, 8, 11, 18, and 21 years. Partners completed EPDS questionnaires once during pregnancy (at 18 weeks’ gestation) and when children were approximately aged 8 weeks; aged 8, 21, and 33 months; and aged 5, 6, 8, 11, and 21 years. Our analyses included biological fathers only.

We focused our primary analyses on parental depression as the exposure because it was available from pregnancy through early adulthood. Supplementary analyses included parental anxiety, which was measured from pregnancy through mid-childhood only. Maternal anxiety was assessed using the anxiety subscale of the Crown-Crisp Experiential Index, a self-reported instrument with scores ranging from 0 (low anxiety) to 16 (high anxiety), twice during pregnancy (at 18 weeks’ gestation and 32 weeks’ gestation) and when children were approximately aged 8 weeks; aged 8, 21, and 33 months; and aged 5 and 6 years.^26^ Paternal anxiety was assessed once during pregnancy (at 18 weeks’ gestation) and then concurrent with maternal assessments.

We selected outcomes based on literature reporting the association of parental mental health with offspring depression, anxiety, and psychotic disorders.^1,9,12,27,28^ We also included offspring alcohol use disorder (AUD) based on studies reporting the association between prenatal stress and substance use.^29^ All outcomes were assessed in early adulthood, which coincides with the peak age of onset for such psychiatric disorders.^13^ Specifically, offspring depression was measured at age 27 years using the EPDS.^23^ Offspring anxiety was assessed at age 25 years using the Screen for Adult Anxiety Related Disorders (SCAARED) questionnaire, which includes 44 items, with scores of 23 or more indicating the presence of an anxiety disorder.^30^ Offspring psychotic symptoms were measured using the Psychosis-Like Symptoms Interview at age 24 years. Psychotic experiences are associated with and predictive of psychotic disorders, including schizophrenia.^31^ Interviewers evaluated the presence of 12 types of psychotic experiences in adult offspring, including hallucinations, delusions, and thought insertion, since aged 12 years. Thus, we considered parental depression exposure from pregnancy through offspring at age 11 years for these analyses. Interviewers rated psychotic experiences as not present, suspected, or definitely present.^32^ We excluded psychotic symptoms related to sleep or fever. Offspring hazardous alcohol use was measured at age 22 years using the Alcohol Use Disorder Identification Test (AUDIT), a 10-item screening instrument for problematic alcohol use, with each item scored from 0 to 4, in which higher scores indicate harmful alcohol use.^33^

We adjusted for confounders during pregnancy that were likely to be associated with parental and offspring mental health including measures associated with socioeconomic status, parity, and parental ages. We did not include child race and ethnicity (because it was self-reported by approximately 98% as White) or maternal-reported prenatal use of anti-anxiety (1%) and anti-depressant (1%) medications. To avoid conditioning on potential mediators, measures of parental prenatal smoking, alcohol use, and obstetric outcomes were considered in secondary analyses.

Genetic factors can confound associations between parental depression and offspring mental health.^2,34^ Polygenic Risk Scores (PRS) are weighted summary scores that capture genetic risk for polygenic phenotypes using common genetic variants (ie, single nucleotide polymorphisms). We used maternal and offspring genotypes and PRS-CSx (Polygenic Risk Score-Continuous Shrinkage [GitHub respository])^35^ (eMethods in Supplement 1) to calculate PRS for major depressive disorder (MDD), generalized anxiety disorder, panic disorder, schizophrenia, bipolar disorder, and AUD, which were included as covariates in all models (paternal genetic data were unavailable).^36,37,38,39,40,41,42^

### Statistical Analysis

Data were analyzed from March 2024 to January 2026. Within the DLM framework, we used proportional odds regression for ordinal outcomes (offspring EPDS, SCAARED, and AUDIT scores) and logistic regression for the presence of psychotic symptoms to calculate the adjusted odds ratio (AOR) of clinically important offspring psychiatric symptoms based on established thresholds (EPDS ≥13, SCAARED ≥23, AUDIT ≥8, and the Psychosis-Like Symptoms Interview = suspected or definite) based on exposure to maternal or paternal symptoms of depression over time.^21,43^ The AOR is defined as the odds of elevated offspring psychiatric symptoms contrasting offspring cumulative exposure to a maternal or paternal EPDS score of 13 vs 0 across all time points. These contrasts and associated AORs are estimated using all available data rather than a comparison of offspring subsets with specific exposure scores. Maternal and paternal depression was considered separately. Exposures at different time points have different weights (importance) that contribute cumulatively to the AOR.^21^ We modeled this association, which captures the importance of the exposure history over time (time-response curve), using penalized P-splines with 15 *df*.

Secondary analyses considered the association between parental anxiety and offspring psychiatric symptoms. We also used distributed lag interaction models and pooled the χ^2^ statistics across imputations using the D2 method to test if offspring sex assigned at birth or offspring PRS modified the cumulative AOR or the time-response curve.^44,45^ We considered nonlinear associations between parental depression and offspring outcomes using a distributed lag nonlinear model (DLNM).^46,47^

Parent-child pairs missing 95% or more of required data were excluded (eMethods in Supplement 1). Missing items within parental EPDS questionnaires were imputed using the multiple correspondence analysis multiple imputation method (missMDA package in R, version 1.20 [R Project for Statistical Computing]), and EPDS total scores and other auxiliary variables were imputed using the random forest multiple imputation method (missRanger package in R, version 2.6.1) (eMethods in Supplement 1).^48,49^ We performed our analyses on 100 imputations of the data and pooled estimates using Rubin rules.^50^ The threshold for statistical significance was 2-sided *P* = .05.

## Results

A total of 5329 adult offspring ALSPAC participants (3276 females [61.5%] and 2053 males [38.5%]) provided at least 1 outcome measure. Measures of depression were available on 3795 participants (mean [SD] age, 27.8 [0.5] years). Symptoms of anxiety were available on 3505 participants (mean [SD] age, 25.3 [0.6] years), while 3342 participants (mean [SD] age, 24.5 [0.8] years) completed interviews to assess risk of psychotic disorders. Symptoms of AUD were available on 3392 participants (mean [SD] age, 22.9 [0.5] years). The Table provides demographic information for those included and excluded from the primary analyses. Offspring symptoms of depression and anxiety were moderately correlated, with weaker correlations observed between other outcomes (eFigure 1 in Supplement 1). Parental EPDS scores are summarized in eTables 1 and 2 in Supplement 1. Correlations between parental depression symptoms across time points are shown in eFigure 2 in Supplement 1.

**Table.** Cohort Demographics^a^

Characteristic	Offspring participants, No. (%)				
	Depression symptoms (EPDS^b^) (n = 3795)	Anxiety symptoms (SCAARED^c^) (n = 3505)	Psychotic experiences (PLIKSi^d^) (n = 3342)	AUD symptoms (AUDIT^e^) (n = 3392)	Exclusions (n = 7245)
Sex					
Female	2506 (66.0)	2314 (66.0)	2086 (62.4)	2203 (64.9)	2844 (39.3)
Male	1289 (34.0)	1191 (34.0)	1256 (37.6)	1189 (35.1)	4401 (60.7)
Maternal age, y					
Mean (SD)	29 (4.6)	29 (4.6)	29 (4.5)	29 (4.4)	27 (5.0)
Missing	11 (0.3)	13 (0.4)	13 (0.4)	14 (0.4)	76 (1.0)
Paternal age, y					
Mean (SD)	31 (5.5)	31 (5.5)	32 (5.4)	32 (5.4)	30 (6.0)
Missing	112 (3.0)	110 (3.1)	103 (3.1)	94 (2.8)	726 (10.0)
Maternal educational level (highest)					
CSE	400 (10.5)	377 (10.8)	293 (8.8)	329 (9.7)	1684 (23.2)
Vocational	288 (7.6)	253 (7.2)	241 (7.2)	232 (6.8)	721 (10.0)
O level^f^	1288 (33.9)	1130 (32.2)	1113 (33.3)	1135 (33.5)	2217 (30.6)
A level^g^	993 (26.2)	953 (27.2)	935 (28.0)	942 (27.8)	1190 (16.4)
Degree	702 (18.5)	675 (19.3)	665 (19.9)	661 (19.5)	517 (7.1)
Missing	124 (3.3)	117 (3.3)	95 (2.8)	93 (2.7)	916 (12.6)
Paternal educational level (highest)					
CSE	638 (16.8)	554 (15.8)	511 (15.3)	514 (15.2)	1956 (27.0)
Vocational	256 (6.7)	243 (6.9)	222 (6.6)	204 (6.0)	570 (7.9)
O level^f^	732 (19.3)	671 (19.1)	660 (19.7)	665 (19.6)	1303 (18.0)
A level^g^	1049 (27.6)	972 (27.7)	933 (27.9)	961 (28.3)	1440 (19.9)
Degree	935 (24.6)	881 (25.1)	867 (25.9)	891 (26.3)	730 (10.1)
Missing	185 (4.9)	184 (5.2)	149 (4.5)	157 (4.6)	1246 (17.2)

Abbreviations: A level, advanced level; AUD, alcohol use disorder; AUDIT, AUD Identification Test; CSE, Certificate of Secondary Education; EPDS, Edinburgh Postnatal Depression Scale; O level, ordinary level; PLIKSi, Psychosis-Like Symptoms Interview; SCAARED, Screen for Adult Anxiety Related Disorders.

^a^ Characteristics of the study sample are grouped by availability of offspring mental health outcomes. Parental educational level at the time of pregnancy was recorded based on the education system in the UK.

^b^ Scores range from 0 to 30, with higher scores indicating more severe depressive symptoms.

^c^ Includes 44 items, with scores of 23 or more indicating the presence of an anxiety disorder.

^d^ Based on interviewer rating for psychotic experiences as being definitely present, suspected, or not present.

^e^ A 10-item screening instrument for problematic alcohol use, with each item scored from 0 to 4, in which higher scores indicate harmful alcohol use.

^f^ Standardized test typically taken at age 16 years, often required for further education or vocational training.

^g^ Standardized test typically taken at ages 17 or 18 years for university admissions and recognized internationally as a qualification for higher education.

### Associations Between Parental Depression and Offspring Mental Health in Adulthood

Using the DLM, we estimated the association between elevated symptoms of parental depression (EPDS = 13) across all time points and offspring depressive symptoms in adulthood. Offspring with exposure to maternal depression across all time points were 2.36 (95% CI, 1.91-2.92) times more likely to exhibit high levels of depressive symptoms when compared with offspring born to mothers without depressive symptoms (EPDS = 0). Those exposed to paternal depression (EPDS = 13) across all time points were 2.13 (95% CI, 1.60-2.83) times more likely to exhibit high levels of depression.

**Figure 1.**
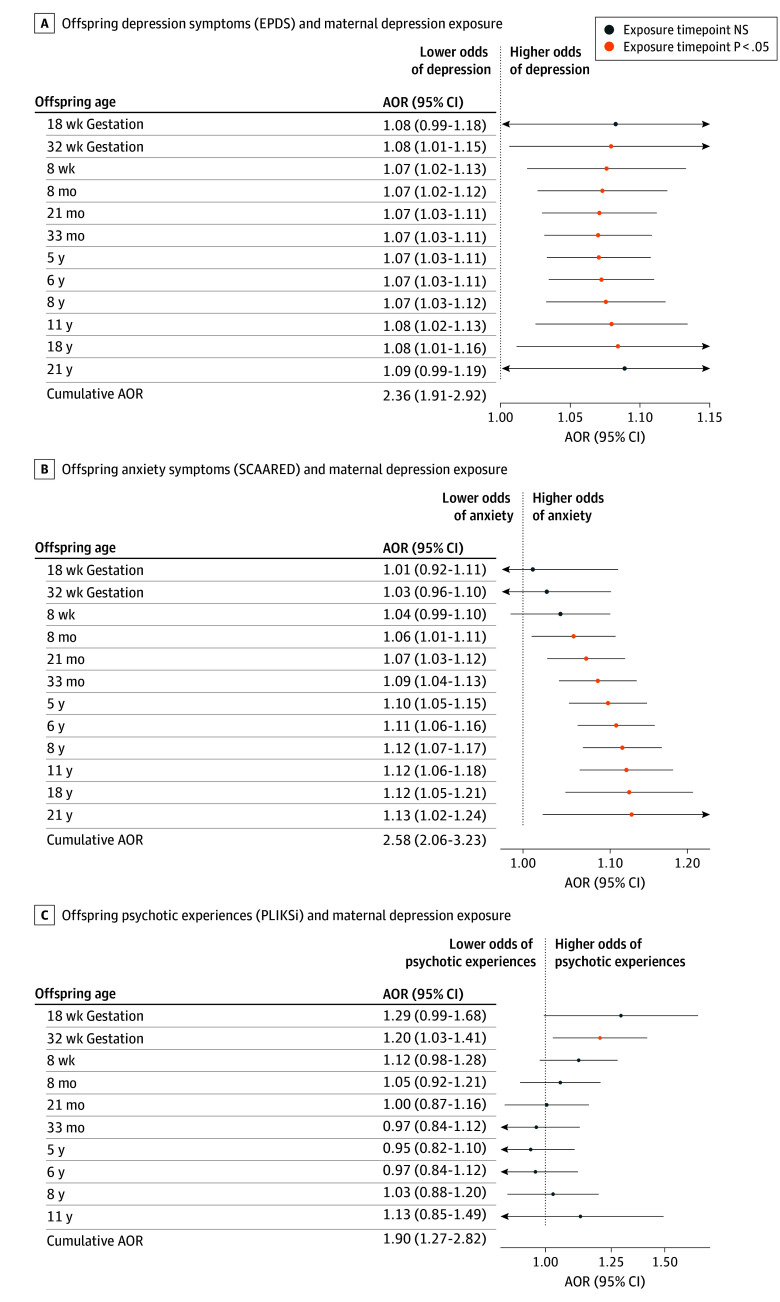
Time-Response Plots of Developmental Exposure to Maternal Depression and Adult Mental Health The adjusted odds ratio (AOR) is defined as the odds of offspring reporting clinically significant symptoms by contrasting exposure to a maternal Edinburgh Postnatal Depression Scale (EPDS) score of 13 across all measured time points vs no exposure over time (ie, maternal EPDS = 0), in which scores range from 0 to 30, with higher scores indicating more severe depressive symptoms. Clinically significant offspring symptoms were defined by an EPDS of 13 or more for depression (A); a Screen for Adult Anxiety Related Disorders (SCAARED) of 23 or more for anxiety, which includes 44 items, with scores of 23 or more indicating the presence of an anxiety disorder (B); and the Psychosis-Like Symptoms Interview (PLIKSi) based on interviewer rating for psychotic experiences as being definitely present, suspected, or not present (C). NS indicates not significant.

**Figure 2.**
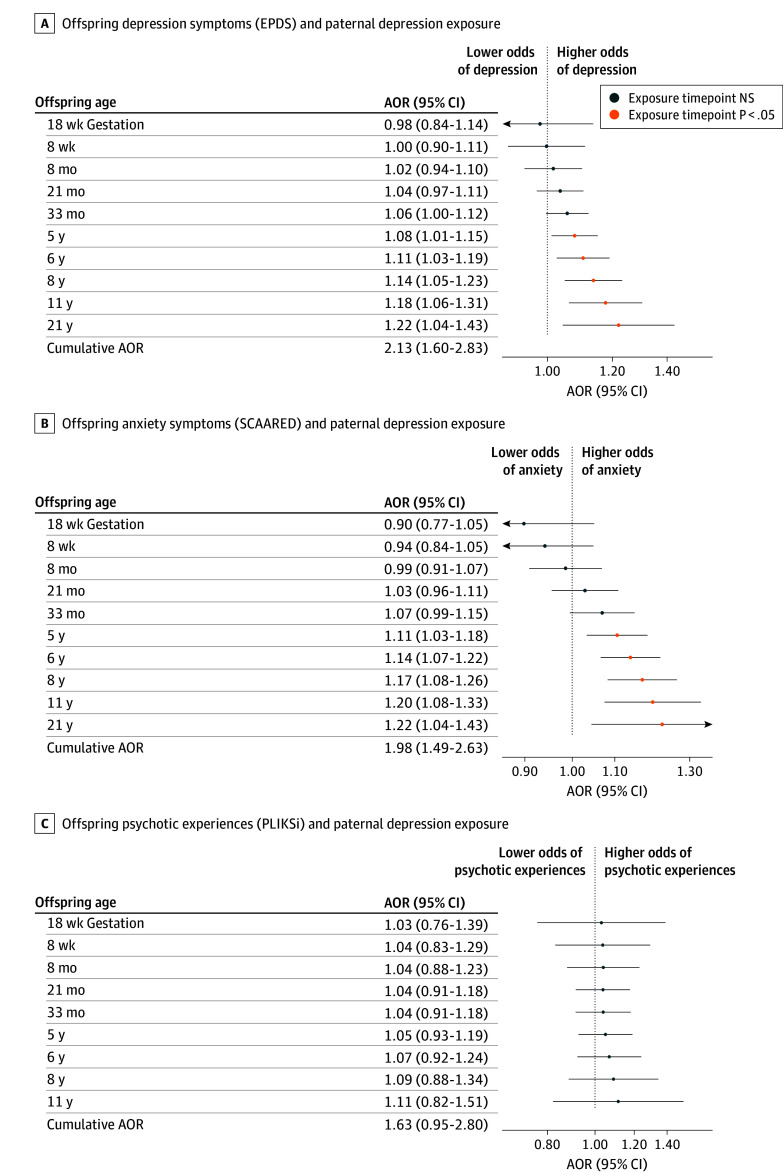
Time-Response Plots of Developmental Exposure to Paternal Depression and Adult Mental Health The adjusted odds ratio (AOR) is defined as the odds of offspring reporting clinically significant symptoms by contrasting exposure to a paternal Edinburgh Postnatal Depression Scale (EPDS) score of 13 across all measured time points vs no exposure over time (ie, paternal EPDS = 0), in which scores range from 0 to 30, with higher scores indicating more severe depressive symptoms. Clinically significant offspring symptoms were defined by an EPDS of 13 or more (A); a Screen for Adult Anxiety Related Disorders (SCAARED) of 23 or more, which includes 44 items, with scores of 23 or more indicating the presence of an anxiety disorder, for anxiety (B); and the Psychosis-Like Symptoms Interview (PLIKSi) based on interviewer rating for psychotic experiences as being definitely present, suspected, or not present (C). NS indicates not significant.

For every exposure time point from 32 weeks’ gestation (AOR, 1.08 [95% CI, 1.01-1.15]) to age 18 years (AOR, 1.08 [95% CI, 1.01-1.16]), maternal depression was significantly associated with increased odds of offspring depression. The shape of the time-response curve remained stable over time. For fathers, depressive symptoms from age 5 years (AOR, 1.08 [95% CI, 1.01-1.15]) onward (aged 21 years: AOR, 1.22 [95% CI, 1.04-1.43]) were significantly associated with offspring depression. The association between paternal and offspring depression increased over time, peaking at 21 years (Figure 1A and Figure 2A). Higher offspring PRS for MDD, lower paternal social class, lower levels of maternal education, and female sex significantly increased the odds of offspring depression symptoms (eTables 3 and 4 in Supplement 1).

Offspring exposed to maternal depression across all time points were 2.58 (95% CI, 2.06-3.23) times more likely to report high levels of anxiety symptoms. Offspring exposed to paternal depression over time were 1.98 times (95% CI, 1.49-2.63) more likely to experience high anxiety levels.

Exposure to maternal depression from age 8 months’ postpartum (AOR, 1.06 [95% CI, 1.01-1.11]) onward (aged 21 years: AOR, 1.13 [95% CI, 1.02-1.24]) was significantly associated with offspring anxiety. The association gradually increased until 8 years of age (AOR, 1.12 [95% CI, 1.07-1.17]) and then remained relatively stable. Exposure to paternal depression was significantly associated with offspring anxiety from 5 years of age (AOR, 1.11 [95% CI, 1.03-1.18]) onward (aged 21 years: AOR, 1.22 [95% CI, 1.04-1.43]) (Figure 1B and Figure 2B). Higher offspring MDD PRS, increased household crowding, lower levels of maternal education, and offspring female sex were associated with higher odds of offspring anxiety (eTables 5 and 6 in Supplement 1).

Adults exposed to maternal depression throughout development were 1.90 (95% CI, 1.27-2.82) times more likely to report psychotic symptoms; paternal depression was not significantly associated with offspring psychotic symptoms (AOR, 1.63 [95% CI, 0.95-2.80]; *P* = .08). The time-response curve shows that only maternal depression at 32 weeks’ gestation (AOR, 1.20 [95% CI, 1.03-1.41]) was associated with higher odds of offspring psychotic symptoms (Figure 1C and Figure 2C). Lower maternal social class, primiparity, being unmarried, offspring male sex, and household crowding were associated with elevated psychotic symptoms (eTables 7 and 8 in Supplement 1). Neither maternal nor paternal depression was significantly associated with offspring problematic alcohol use at age 22 years (EPDS maternal: AOR, 1.12 [95% CI, 0.90-1.41]; paternal: AOR, 1.13 [95% CI, 0.84-1.53]) (eFigure 3 and eTables 9 and 10 in Supplement 1).

### Secondary Analyses

Results from models that additionally adjusted for prenatal parental smoking, alcohol use, birth weight, and gestational age were consistent with our primary analyses (eFigure 4 in Supplement 1). We found no evidence for moderation by offspring sex or polygenic risk for psychiatric disorders (eTables 11 and 12 in Supplement 1).

**Figure 3.**
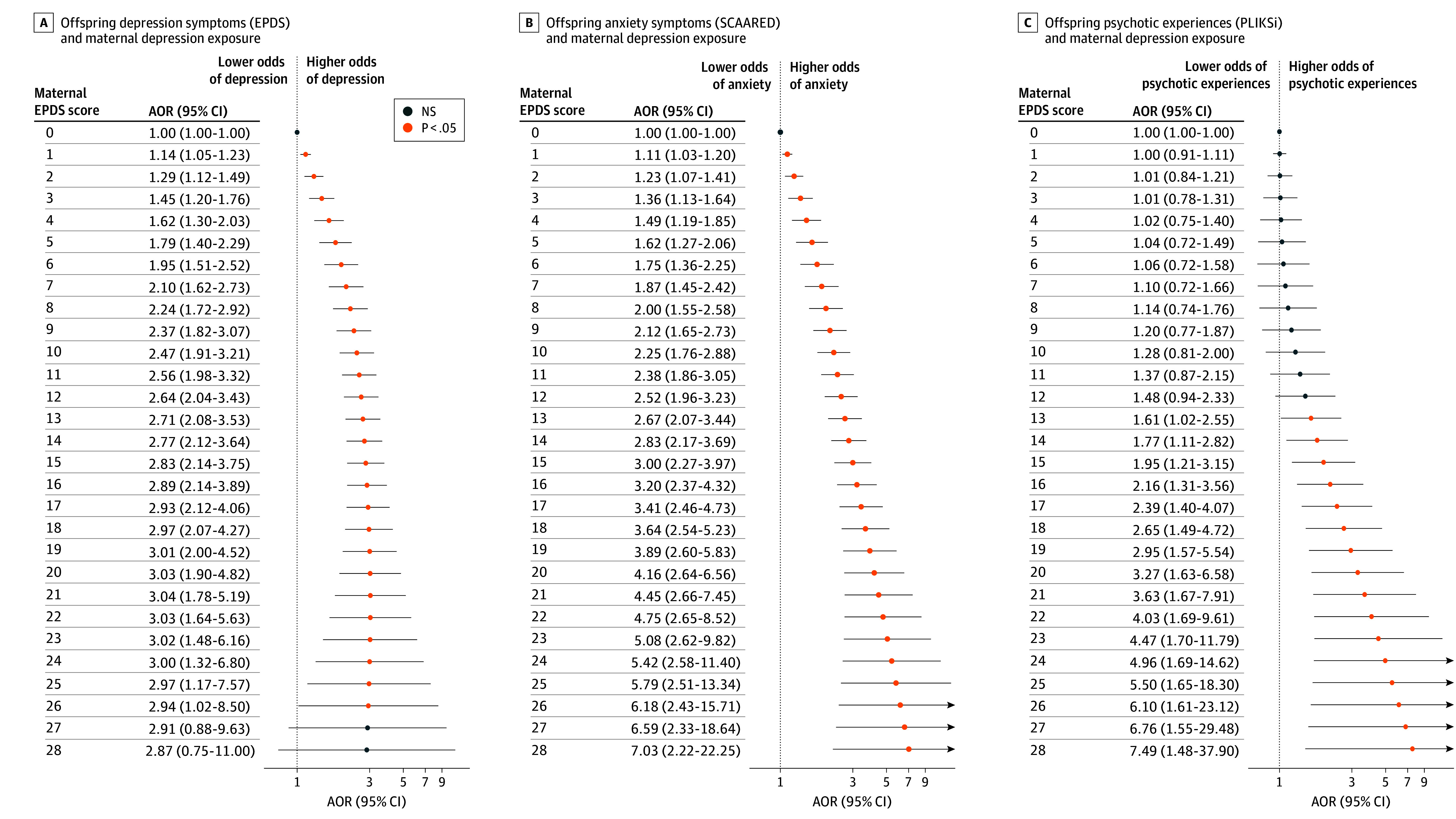
Time-Response Plots of Nonlinear Associations Between Maternal Depression Exposure and Adult Mental Health Distributed lag nonlinear models were used to examine potential nonlinear associations between maternal Edinburgh Postnatal Depression Scale (EPDS) scores and offspring mental health outcomes. The adjusted odds ratio (AOR) of clinically significant symptoms represents contrasts between maternal EPDS scores across a range of observed values (from 1-30) vs a maternal EPDS score of 0. Clinically significant offspring symptoms were defined by an EPDS of 13 or more for depression (A); a Screen for Adult Anxiety Related Disorders (SCAARED) of 23 or more, which includes 44 items, with scores of 23 or more indicating the presence of an anxiety disorder, for anxiety (B); and the Psychosis-Like Symptoms Interview (PLIKSi) based on interviewer rating for psychotic experiences as being definitely present, suspected, or not present (C). NS indicates not significant.

**Figure 4.**
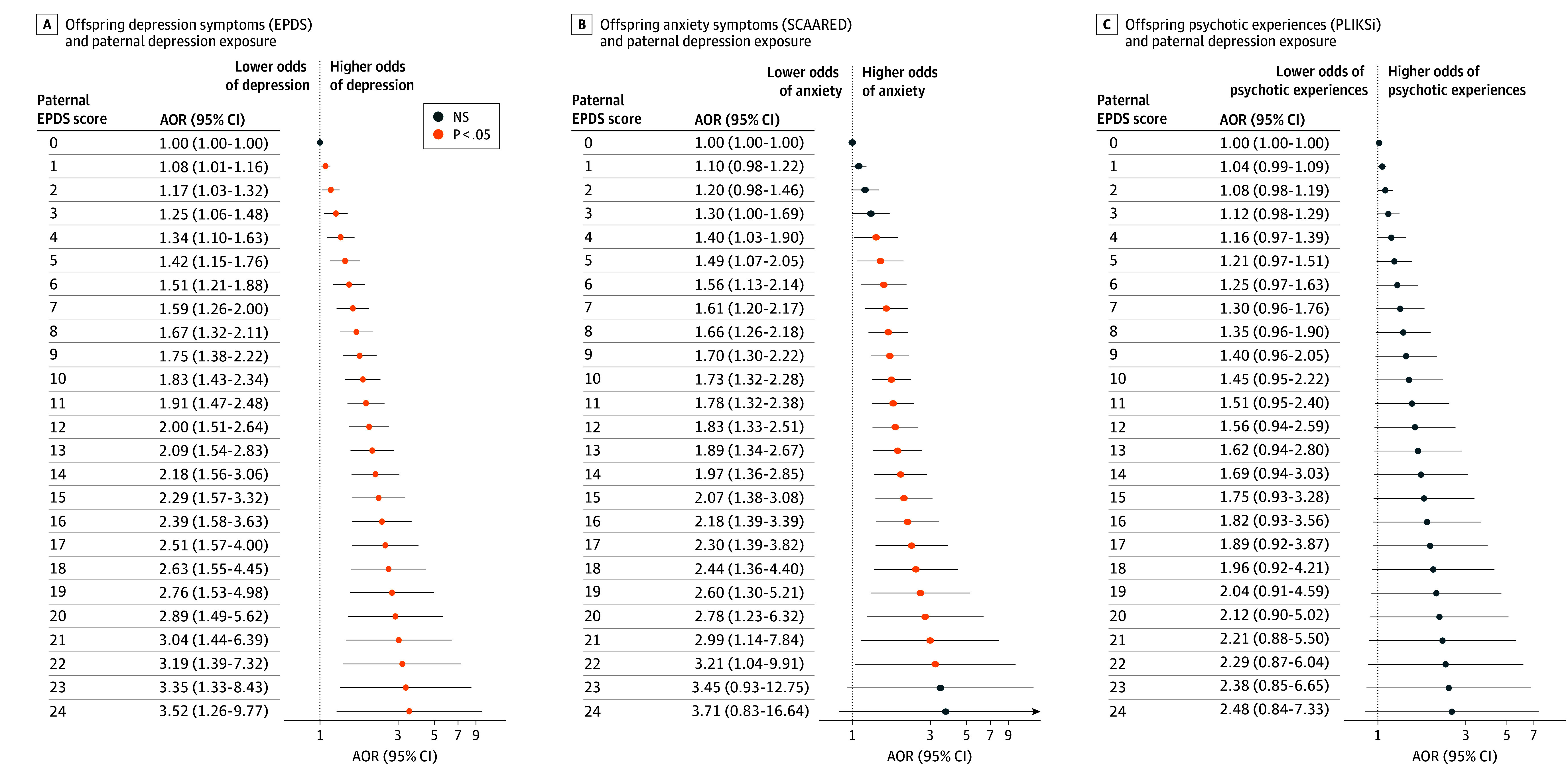
Time-Response Plots of Nonlinear Associations Between Paternal Depression Exposure and Adult Mental Health Distributed lag nonlinear models were used to examine potential nonlinear associations between paternal Edinburgh Postnatal Depression Scale (EPDS) scores and offspring mental health outcomes. The adjusted odds ratio (AOR) of clinically significant symptoms represents contrasts between paternal EPDS scores across a range of observed values (from 1-30) vs a paternal EPDS score of 0. Clinically significant offspring symptoms were defined by an EPDS of 13 or more for depression (A); a Screen for Adult Anxiety Related Disorders (SCAARED) of 23 or more, which includes 44 items, with scores of 23 or more indicating the presence of an anxiety disorder, for anxiety (B); and the Psychosis-Like Symptoms Interview (PLIKSi) based on interviewer rating for psychotic experiences as being definitely present, suspected, or not present (C). NS indicates not significant.

Nonlinear models (DLNMs) found significant associations between maternal depression across almost the entire range of symptom severity and offspring depression and anxiety (Figure 3A and B). A nonlinear pattern can be observed between maternal depression and offspring symptoms of psychotic disorders, which was statistically significant at higher maternal symptom levels only (EPDS ≥13) (Figure 3C). Paternal EPDS scores showed an approximately linear pattern of association with offspring depression and anxiety, but no association was observed with psychotic experiences (Figure 4). The time-response curves for these nonlinear models (DLNMs) closely resembled those from linear models (DLMs) (eFigure 5 in Supplement 1). Distinct temporal associations emerged between maternal and paternal anxiety (eTables 13 and 14 in Supplement 1) and each adult mental health outcome studied but with patterns of associations that were consistent with that of parental depression (eFigure 6 and eTables 15-22 in Supplement 1).

## Discussion

In this cohort study, we leveraged measures of parental mental health across more than 2 decades of life paired with direct assessments of offspring symptoms of depression, anxiety, psychotic disorders, and AUD to examine developmental timing effects in the association between maternal and paternal depression and multiple domains of mental health in adulthood. We observed exposure time-dependent associations between maternal and paternal depression and each adult mental health outcome, with evidence of pregnancy as a sensitive period of exposure for maternal depression and adult psychotic experiences. These associations, which included maternal and offspring PRS for multiple psychiatric disorders and exposure periods from pregnancy, suggest a role of timing between exposure to parental depression and offspring mental health.

Maternal depression at almost every time point in development, from pregnancy onward, was significantly associated with offspring symptoms of depression—to our knowledge, a novel finding in the literature, which has considered far more limited exposure periods and often neglected the prenatal period. Similarly, from 8 months’ postpartum onward, maternal depression was associated with offspring anxiety symptoms. Only maternal depression in late pregnancy was significantly associated with offspring symptoms of psychosis. In contrast, paternal prenatal depression was not significantly associated with any psychiatric outcome in this study. Rather, exposure to paternal depression from mid-childhood onward (aged 5 years) was associated with both offspring symptoms of depression and anxiety in adulthood. These exposure time-dependent findings point to the presence of multiple and distinct mechanisms underlying the association between parental depression and adult psychiatric symptoms. The biological connection between mother and child in utero may explain, at least in part, the association between maternal but not paternal prenatal depression and high offspring psychiatric symptom burden.

There is considerable interest in the degree to which there may be differential or synergistic effects of maternal and paternal influences on offspring mental health, whether related to genetic risk or parenting.^51^ The difference between maternal and paternal timing effects in models that include genetic risk for psychiatric disorders suggests that these associations are not easily accounted for by simple vertical transmission of genetic risk.^12,52^ It is important to note that despite differences in effect sizes at specific time points, the cumulative associations between maternal and paternal depression and high offspring psychiatric symptoms were similar, underscoring the need to incorporate both maternal and paternal exposures in life course analyses of adult psychiatric outcomes.

In line with previous studies in childhood and adolescence, we found that chronic exposure to parental depressive symptoms was associated with increased cumulative odds for elevated symptoms of depression, anxiety, and psychosis in adulthood.^1,5,8,9,10,12,27,53,54^ In contrast, we found no evidence of an association between parental depression and problematic alcohol use (AUDIT ≥8). The AUDIT assesses risk across 3 domains: alcohol consumption, dependence, and associated harms.^33^ Our focus on AUDIT total scores may underlie the observed null findings; 1 previous study suggests a nuanced association between parental depression and specific domains of problematic alcohol use.^55^ We found no evidence that offspring sex modifies the association between parental depression and offspring mental health, which is an important nonfinding in a large sample given the inconsistent clinical findings and despite compelling evidence from preclinical studies.^1,11,12,56,57,58,59^

Our primary analyses support a linear dose-response association between parental depressive symptoms and offspring symptoms of anxiety and depression. However, the nonlinear models suggest that an association between maternal depression and offspring symptoms of psychotic experiences was only observed at higher symptom levels. This finding is consistent with epidemiologic studies that found prenatal exposure to severe maternal stress was associated with psychotic illnesses in adult offspring.^60,61,62,63,64,65^

Human brain development encompasses spatiotemporal changes in gene expression, neurogenesis and cell migration, synaptogenesis, synaptic pruning, and myelination.^66^ Each of these processes, which have distinct developmental profiles, may underlie the clinical outcomes assessed in the current study as well as previous reports of exposure time-dependent associations between maternal prenatal and postnatal depression or stress and offspring brain structure.^67,68^ The complex interplay between these mechanisms of early brain development does not lead easily to hypotheses about exposure timing, but the specific association between prenatal maternal depression and adult psychotic symptoms is consistent with a potential mechanism involving synaptogenesis, which peaks in utero.^69,70^

### Strengths and Limitations

This study has several strengths. Our study considered both maternal and paternal symptoms with assessments spanning pregnancy through early adulthood. Our measures of offspring mental health encompassed multiple symptom domains in adulthood, beyond the peak age of onset for most psychiatric illnesses, and were assessed directly via self-report, extending existing evidence that the likelihood of certain adult psychiatric illnesses increases with chronic exposure to parental depression and is not explained by reporter bias.^13,14,71,72,73,74,75^ Our imputation approach (eMethods in Supplement 1) maximized the power of our analysis and mitigated potential sources of bias. Furthermore, the DLM provides precise estimates despite the moderate amount of multicollinearity observed between consecutive measures of parental depression.

Some limitations of this study are important to note. Almost all mothers and children self-identified as White, reflecting the demographics of the community from which the cohort was sampled at that time, which may limit the generalizability of our results. There was also selective attrition of male participants and participants born to parents reporting high depressive symptoms. This pattern of missing data may have decreased our power to detect sex differences in the association between parental depression and offspring mental health. Although our models considered both maternal and child PRS for relevant psychiatric disorders, offspring copy number variants or rare variants were not considered. The absence of paternal genetic data was a limitation of our study and a source of potential residual genetic confounding.^2,16,17,18^ Additionally, the observational nature of this cohort study precludes causal inference. Evidence-based interventions that target parental mental health and assess offspring psychiatric outcomes are another important future direction.^76,77^

## Conclusions

In this cohort study, exposure to parental depressive symptoms across the life course, from pregnancy to young adulthood, was associated with higher odds of depression, anxiety, and psychotic disorders in adulthood. However, the exposure timing effects were different for mothers and fathers, pointing to distinct underlying mechanisms. Our findings underline the importance of screening and treating mental illness in both parents, not only during the prenatal period but also throughout childhood. Our findings also suggest that reduced exposure to parental depression may have secondary, positive effects across multiple symptom domains in the next generation.

## Notes

Supplement 1.
eMethods.
**eTable 1.** Descriptive Statistics for Maternal Edinburgh Postnatal Depression Scale (EPDS) Scores (0-30) From 18 Weeks’ Gestation to 21 Years, Stratified by Outcome Variables **eTable 2.** Descriptive Statistics for Paternal Edinburgh Postnatal Depression Scale (EPDS) Scores (0-30) From 18 Weeks’ Gestation to 21 Years, Stratified by Outcome Variables **eTable 3.** Regression Table Between Maternal Edinburgh Postnatal Depression Scale (EPDS) and Offspring Age 27 EPDS Scores **eTable 4.** Regression Table Between Paternal Edinburgh Postnatal Depression Scale (EPDS) and Offspring Age 27 EPDS Scores **eTable 5.** Regression Table Between Maternal Edinburgh Postnatal Depression Scale (EPDS) and Offspring Age 25 Screen for Adult Anxiety Related Disorders (SCAARED) Score **eTable 6.** Regression Table Between Paternal Edinburgh Postnatal Depression Scale (EPDS) and Offspring Age 25 Screen for Adult Anxiety Related Disorders (SCAARED) Score **eTable 7.** Regression Table Between Maternal Edinburgh Postnatal Depression Scale (EPDS) and Offspring “Definite” or “Suspected” Psychotic Experiences From 12-24 (Measured at 24 Using the Psychotic-Like Symptoms Interview [PLIKSi] Interview) **eTable 8.** Regression Table Between Paternal Edinburgh Postnatal Depression Scale (EPDS) and Offspring “Definite” or “Suspected” Psychotic Experiences From 12-24 (Measured at 24 Using the Psychotic-Like Symptoms Interview [PLIKSi] Interview) **eTable 9.** Regression Table Between Maternal Edinburgh Postnatal Depression Scale (EPDS) and Adult Offspring Alcohol Use Disorder Identification Test (AUDIT) Score **eTable 10.** Regression Table Between Paternal Edinburgh Postnatal Depression Scale (EPDS) and Adult Offspring Alcohol Use Disorder Identification Test (AUDIT) Score **eTable 11.** Chi-Square Tests for Goodness of Fit Between the Distributed Lag Interaction Model (DLIM) (With Sex as the Modifying Variable) and the Distributed Lag Model (DLM) for Each Parental Mental Health Exposure—Offspring Mental Health Outcome Pair **eTable 12.** Chi-Square Tests for Goodness of Fit Between the Distributed Lag Interaction Model (DLIM) (With PRS as the Modifying Variable) and the Distributed Lag Model (DLM) for Each Parental Mental Health Exposure—Offspring Mental Health Outcome Pair **eTable 13.** Descriptive Statistics for Maternal Crown-Crisp Experiential Index (CCEI) Anxiety Subscores (0-16) From 18 Weeks’ Gestation to 6 Years, Stratified by Outcome Variables **eTable 14.** Descriptive Statistics for Paternal Crown-Crisp Experiential Index (CCEI) Anxiety Subscores (0-16) From 18 Weeks’ Gestation to 6 Years, Stratified by Outcome Variables **eTable 15.** Regression Table Between Maternal Crown-Crisp Experiential Index (CCEI) Anxiety Subscores and Offspring Age 27 EPDS Scores **eTable 16.** Regression Table Between Paternal Crown-Crisp Experiential Index (CCEI) Anxiety Subscores and Offspring Age 27 EPDS Scores **eTable 17.** Regression Table Between Maternal Crown-Crisp Experiential Index (CCEI) Anxiety Subscores and Offspring Age 25 Screen for Adult Anxiety Related Disorders (SCAARED) Score **eTable 18.** Regression Table Between Paternal Crown-Crisp Experiential Index (CCEI) Anxiety Subscores and Offspring Age 25 Screen for Adult Anxiety Related Disorders (SCAARED) Score **eTable 19.** Regression Table Between Maternal Crown-Crisp Experiential Index (CCEI) Anxiety Subscores and Offspring “Definite” or “Suspected” Psychotic Experiences From 12-24 (Measured at 24 Using the Psychotic-Like Symptoms Interview [PLIKSi] Interview) **eTable 20.** Regression Table Between Paternal Crown-Crisp Experiential Index (CCEI) Anxiety Subscores and Offspring “Definite” or “Suspected” Psychotic Experiences From 12-24 (Measured at 24 Using the Psychotic-Like Symptoms Interview [PLIKSi] Interview) **eTable 21.** Regression Table Between Maternal Crown-Crisp Experiential Index (CCEI) Anxiety Subscores and Adult Offspring Alcohol Use Disorder Identification Test (AUDIT) Score **eTable 22.** Regression Table Between Paternal Crown-Crisp Experiential Index (CCEI) Anxiety Subscores and Adult Offspring Alcohol Use Disorder Identification Test (AUDIT) Score **eFigure 1.** Marginal Distributions and Correlations Between Adult Mental Health Outcomes **eFigure 2.** Correlation Plots of Maternal and Paternal Edinburgh Postnatal Depression Scale (EPDS) Scores and Crown-Crisp Experiential Index (CCEI) Anxiety Subscores Across Multiple Measured Time Points From 18 Weeks’ Gestation to 21 Years of Age (for EPDS) and From 18 Weeks’ Gestation to 6 Years of Age (for CCEI) **eFigure 3.** Adjusted Odds Ratio (AOR) and Longitudinal Effect of Parental EPDS on Adult Offspring Alcohol Use Disorder Identification Test (AUDIT) Score **eFigure 4.** Adjusted Odds Ratio (AOR) and Longitudinal Effect of Parental EPDS on Adult Offspring Measures of Mental Health, Distributed Lag Model Additionally Adjusted for Gestational Age, Birth Weight, Prenatal Smoking, and Alcohol Use **eFigure 5.** Longitudinal Effect of Parental EPDS on Adult Offspring Measures of Mental Health Using the Distributed Lag Nonlinear Models (DLNMs) **eFigure 6.** Adjusted Odds Ratio (AOR) and Longitudinal Effect of Parental Crown-Crisp Experiential Index (CCEI) Anxiety Subscores on Adult Offspring Measures of Mental Health
eReferences

Supplement 2.
Data Sharing Statement
