# Supplementary material for: Timing of Exposure to Parental Depression From Pregnancy to Young Adulthood and Mental Health in Adult Offspring
Source: JAMA Netw Open. 2026 Apr 10;9(4):e264892. doi: 10.1001/jamanetworkopen.2026.4892 (PMC13069460; doi:10.1001/jamanetworkopen.2026.4892)
Supplement: Supplement 1. — eMethods. eTable 1. Descriptive Statistics for Maternal Edinburgh Postnatal Depression Scale (EPDS) Scores (0-30) From 18 Weeks’ Gestation to 21 Years, Stratified by Outcome Variables eTable 2. Descriptive Statistics for Paternal Edinburgh Postnatal Depression Scale (EPDS) Scores (0-30) From 18 Weeks’ Gestation to 21 Years, Stratified by Outcome Variables eTable 3. Regression Table Between Maternal Edinburgh Postnatal Depression Scale (EPDS) and Offspring Age 27 EPDS Scores eTable 4. Regression Table Between Paternal Edinburgh Postnatal Depression Scale (EPDS) and Offspring Age 27 EPDS Scores eTable 5. Regression Table Between Maternal Edinburgh Postnatal Depression Scale (EPDS) and Offspring Age 25 Screen for Adult Anxiety Related Disorders (SCAARED) Score eTable 6. Regression Table Between Paternal Edinburgh Postnatal Depression Scale (EPDS) and Offspring Age 25 Screen for Adult Anxiety Related Disorders (SCAARED) Score eTable 7. Regression Table Between Maternal Edinburgh Postnatal Depression Scale (EPDS) and Offspring “Definite” or “Suspected” Psychotic Experiences From 12-24 (Measured at 24 Using the Psychotic-Like Symptoms Interview [PLIKSi] Interview) eTable 8. Regression Table Between Paternal Edinburgh Postnatal Depression Scale (EPDS) and Offspring “Definite” or “Suspected” Psychotic Experiences From 12-24 (Measured at 24 Using the Psychotic-Like Symptoms Interview [PLIKSi] Interview) eTable 9. Regression Table Between Maternal Edinburgh Postnatal Depression Scale (EPDS) and Adult Offspring Alcohol Use Disorder Identification Test (AUDIT) Score eTable 10. Regression Table Between Paternal Edinburgh Postnatal Depression Scale (EPDS) and Adult Offspring Alcohol Use Disorder Identification Test (AUDIT) Score eTable 11. Chi-Square Tests for Goodness of Fit Between the Distributed Lag Interaction Model (DLIM) (With Sex as the Modifying Variable) and the Distributed Lag Model (DLM) for Each Parental Mental Health Exposure—Offspring Mental Health Outcome Pair eTa [file jamanetwopen-e264892-s001.pdf]

Supplemental Online Content

Feibel A, Pham H, Glover V, O'Connor TG, O'Donnell KJ. Timing of exposure to parental depression from pregnancy to young adulthood and mental health in adult offspring. *JAMA Netw Open*. 2026;9(4):e264892. doi:10.1001/jamanetworkopen.2026.4892

eMethods

eTable 1. Descriptive Statistics for Maternal Edinburgh Postnatal Depression Scale (EPDS) Scores (0-30) From 18 Weeks' Gestation to 21 Years, Stratified by Outcome Variables

eTable 2**.** Descriptive Statistics for Paternal Edinburgh Postnatal Depression Scale (EPDS) Scores (0-30) From 18 Weeks' Gestation to 21 Years, Stratified by Outcome Variables

eTable 3**.** Regression Table Between Maternal Edinburgh Postnatal Depression Scale (EPDS) and Offspring Age 27 EPDS Scores

eTable 4. Regression Table Between Paternal Edinburgh Postnatal Depression Scale (EPDS) and Offspring Age 27 EPDS Scores

eTable 5. Regression Table Between Maternal Edinburgh Postnatal Depression Scale (EPDS) and Offspring Age 25 Screen for Adult Anxiety Related Disorders (SCAARED) Score

eTable 6. Regression Table Between Paternal Edinburgh Postnatal Depression Scale (EPDS) and Offspring Age 25 Screen for Adult Anxiety Related Disorders (SCAARED) Score

eTable 7. Regression Table Between Maternal Edinburgh Postnatal Depression Scale (EPDS) and Offspring "Definite" or "Suspected" Psychotic Experiences From 12-24 (Measured at 24 Using the Psychotic-Like Symptoms Interview [PLIKSi] Interview)

eTable 8. Regression Table Between Paternal Edinburgh Postnatal Depression Scale (EPDS) and Offspring "Definite" or "Suspected" Psychotic Experiences From 12-24 (Measured at 24 Using the Psychotic-Like Symptoms Interview [PLIKSi] Interview)

eTable 9. Regression Table Between Maternal Edinburgh Postnatal Depression Scale (EPDS) and Adult Offspring Alcohol Use Disorder Identification Test (AUDIT) Score

eTable 10. Regression Table Between Paternal Edinburgh Postnatal Depression Scale (EPDS) and Adult Offspring Alcohol Use Disorder Identification Test (AUDIT) Score

eTable 11. Chi-Square Tests for Goodness of Fit Between the Distributed Lag Interaction Model (DLIM) (With Sex as the Modifying Variable) and the Distributed Lag Model (DLM) for Each Parental Mental Health Exposure—Offspring Mental Health Outcome Pair

eTable 12. Chi-Square Tests for Goodness of Fit Between the Distributed Lag Interaction Model (DLIM) (With PRS as the Modifying Variable) and the Distributed Lag Model (DLM) for Each Parental Mental Health Exposure—Offspring Mental Health Outcome Pair

eTable 13. Descriptive Statistics for Maternal Crown-Crisp Experiential Index (CCEI) Anxiety Subscores (0-16) From 18 Weeks' Gestation to 6 Years, Stratified by Outcome Variables

eTable 14. Descriptive Statistics for Paternal Crown-Crisp Experiential Index (CCEI) Anxiety Subscores (0-16) From 18 Weeks' Gestation to 6 Years, Stratified by Outcome Variables

eTable 15. Regression Table Between Maternal Crown-Crisp Experiential Index (CCEI) Anxiety Subscores and Offspring Age 27 EPDS Scores

eTable 16. Regression Table Between Paternal Crown-Crisp Experiential Index (CCEI) Anxiety Subscores and Offspring Age 27 EPDS Scores

eTable 17. Regression Table Between Maternal Crown-Crisp Experiential Index (CCEI) Anxiety Subscores and Offspring Age 25 Screen for Adult Anxiety Related Disorders (SCAARED) Score

eTable 18. Regression Table Between Paternal Crown-Crisp Experiential Index (CCEI) Anxiety Subscores and Offspring Age 25 Screen for Adult Anxiety Related Disorders (SCAARED) Score

eTable 19. Regression Table Between Maternal Crown-Crisp Experiential Index (CCEI) Anxiety Subscores and Offspring "Definite" or "Suspected" Psychotic Experiences From 12-24 (Measured at 24 Using the Psychotic-Like Symptoms Interview [PLIKSi] Interview)

eTable 20. Regression Table Between Paternal Crown-Crisp Experiential Index (CCEI) Anxiety Subscores and Offspring "Definite" or "Suspected" Psychotic Experiences From 12-24 (Measured at 24 Using the Psychotic-Like Symptoms Interview [PLIKSi] Interview)

© 2026 Feibel A et al. *JAMA Network Open*.

eTable 21. Regression Table Between Maternal Crown-Crisp Experiential Index (CCEI) Anxiety Subscores and Adult Offspring Alcohol Use Disorder Identification Test (AUDIT) Score
eTable 22. Regression Table Between Paternal Crown-Crisp Experiential Index (CCEI) Anxiety Subscores and Adult Offspring Alcohol Use Disorder Identification Test (AUDIT) Score
eFigure 1. Marginal Distributions and Correlations Between Adult Mental Health Outcomes
eFigure 2. Correlation Plots of Maternal and Paternal Edinburgh Postnatal Depression Scale (EPDS) Scores and Crown-Crisp Experiential Index (CCEI) Anxiety Subscores Across Multiple Measured Time Points From 18 Weeks' Gestation to 21 Years of Age (for EPDS) and From 18 Weeks' Gestation to 6 Years of Age (for CCEI)
eFigure 3. Adjusted Odds Ratio (AOR) and Longitudinal Effect of Parental EPDS on Adult Offspring Alcohol Use Disorder Identification Test (AUDIT) Score
eFigure 4. Adjusted Odds Ratio (AOR) and Longitudinal Effect of Parental EPDS on Adult Offspring Measures of Mental Health, Distributed Lag Model Additionally Adjusted for Gestational Age, Birth Weight, Prenatal Smoking, and Alcohol Use
eFigure 5. Longitudinal Effect of Parental EPDS on Adult Offspring Measures of Mental Health Using the Distributed Lag Nonlinear Models (DLNMs)
eFigure 6. Adjusted Odds Ratio (AOR) and Longitudinal Effect of Parental Crown-Crisp Experiential Index (CCEI) Anxiety Subscores on Adult Offspring Measures of Mental Health
eReferences

This supplemental material has been provided by the authors to give readers additional information about their work.

© 2026 Feibel A et al. *JAMA Network Open*.

eMethods

Covariates

We adjusted for confounders related to parental socioeconomic status (highest level of parental education, parental social classes, maternal marital status, and house crowding index) and antenatal characteristics (parity and parental ages).

All maternal and paternal confounders were abstracted from mothers' survey responses to questions about themselves and their partners during pregnancy, and only demographic information from biological parents was included. Education categories ranked from lowest to highest included Certificate of Secondary Education (CSE); vocational training; O-levels (equivalent to modern day General Certificate of Secondary Education); A-levels (comparable to college entrance examinations); and a university degree or higher. Parental social class, which were derived from information about parents' current occupation, were defined using the 1991 British Office of Population and Census Statistics (OPCS) classification as: I (professional occupations), II (managerial and technical occupations), III (non-manual) (skilled occupations (non-manual)), III (manual) (skilled occupations (manual)), IV (partly skilled occupations), and V (unskilled occupations).[1] Marital status categories included first marriage; second or third marriage; never married; and widowed, divorced, or separated. House crowding was derived from the number of residents per room in a household. The four categories included: ≤ 0.5 residents/room; >0.5-0.75; >.75-1; and >1. Parity, or number of prior pregnancies, was factorized into three categories (nulliparous, one previous pregnancy, and two or more previous pregnancies) due to a small number of participants with more than two prior pregnancies.

In a supplemental analysis, we also adjusted for prenatal maternal smoking and alcohol use, birth weight, and gestational age. Maternal smoking was measured by number of cigarettes smoked per day during the first 3 months of pregnancy, which was derived from mothers' survey responses at 18 weeks' gestation. Number of cigarettes smoked per day was defined as four categories: 0, 1-9, and >=10. The last category was factorized due to the low levels of smoking above 9. Prenatal alcohol use was measured by number of alcoholic beverages during first 3 months of pregnancy, which was also derived from mothers' survey responses at 18 weeks' gestation. Number of alcoholic beverages in first 3 months of pregnancy was defined as four categories: never, <1 glass/week, 1+ glass/week, and 1+ glass/day drinkers. The last category was factorized due to the low levels of drinking 1 or more glasses/day. Birth weight and gestational age were abstracted from obstetric medical notes.

Missing Data

We implemented a comprehensive two-step imputation strategy to handle missingness, first at the level of individual questionnaire items, and then at the second level, which included total questionnaire scores with other missing covariates. First, we used Multiple Correspondence Analysis Multiple Imputation (MIMCA) method as implemented in the missMDA package to impute missing data at the item-level (200 imputations).[2] MIMCA is a PCA-based method with favorable performance in imputing sum scores from survey data when all variables (the items) are categorical variables.[2]

For the second level of imputation, we used random forest imputation implemented in the missRanger package to impute missing data for total questionnaire scores and missing covariates.[3] Specifically, we combined each imputation of the sum scores with other analysis variables and auxiliary variables and imputed missing values. Random forest imputation is favorable in maintaining the nonlinear and higher order relationships between variables and can handle different types of data. Similar to the multiple imputation by chained equations (MICE) methods, the random forest imputation imputes the missing values of one variable conditioned on all other variables and iterates through all variables sequentially until the missing values are filled. Additionally, missRanger implements predictive mean matching to generate realistic values and uses the per-variable out-of-bag (OOB) error as a criterion to judge the accuracy of the predictions. We ran the random forest imputation with different seeds and iterated until the OOB error no longer improved and kept the final iterations, resulting in 100 multiple imputations of data. We performed hyperparameter tuning on the following random forest hyperparameters: the number of variables randomly sampled as candidates for each split (mtry), the minimum number of rows required before splitting (min.node.size), the maximum depth of a tree (max.depth), and the total number of trees in the ensemble (num.trees). Our hyperparameter tuning strategy significantly enhanced predictive accuracy across all variables in our imputation model.

© 2026 Feibel A et al. *JAMA Network Open*.

Statistical Analysis

We regressed offspring mental health outcomes on longitudinal measures of parental depressive symptoms, adjusting for covariates, using the distributed lag model (DLM) regression framework and tested if offspring sex modifies this effect using the distributed lag interaction model (DLIM).[4,5] The repeated measures of the exposure were entered into the model as a N*T matrix, where N is the number of individuals included in the data and T is the number of time points, organized from left to right as the most remote to the most recent. Within the DLM framework, we used proportional odds regressions for ordinal outcomes (EPDS, SCAARED, and AUDIT scores) and logistic regression for 'suspected' or 'definite' presence of psychotic experiences (compared to no history of psychotic experiences).

DLM uses a bi-dimensional spline basis expansion between an exposure and its time structure to model the exposure-time-response surface. In formula form, the association is represented as follows. For an exposure value (x), measured at time (t-l), for lag l0 to L, and a vector of covariates (z), the expected value of the outcome (y) is modeled as:

$$g(E[y|x,z]) = s(x,t) + \boldsymbol{\beta}^{\mathsf{T}}\mathbf{z} \quad \approx \sum_{l=l_0}^{L} f\,(x_{t-l}) \circ w(l) + \boldsymbol{\beta}^{\mathsf{T}}\mathbf{z}$$

In this formula, (g) is the link function. The cumulative effect of the exposure history on the outcome is modeled through the function (s(x, t)), which can be parameterized into the summation term. From this parameterization, we observe that (s(x, t)) is comprised of two parts: the exposure-response function (f(xt-l)) and the time-response function (w(l)). The exposure-response function models the association between exposure values and the outcome, and the time-response function models the different weights of the exposure at different time points. In DLM, the exposure-response function is a linear function (f(x) = x).

We then used DLIM, which builds on the DLM framework by studying the exposure-time-response surface at specific values of a third variable, to test if offspring sex modified the exposure-time-response surface.[5] Specifically, Chi-square tests compared the goodness of fit between the Maximum Likelihood (ML) fit of the DLIM and the DLM model. If we rejected the null hypothesis that there was no sex interaction effect, we refitted the DLIM model with sex as a modifier, again using restricted maximum likelihood (REML) and pooling results over 100 imputations.

© 2026 Feibel A et al. *JAMA Network Open*.

eTable 1. Descriptive Statistics for Maternal Edinburgh Postnatal Depression Scale (EPDS) Scores (0-30) From 18 Weeks' Gestation to 21 Years, Stratified by Outcome Variables

	Dep Sx (EPDS) (N=3795)	Anx Sx (SCAARED) (N=3505)	Psych Exp (PLIKS) (N=3342)	AUD Sx (AUDIT) (N=3392)
**Maternal EPDS at 18 Weeks Prenatal**				
Mean (± SD)	6.2 (± 4.5)	6.3 (± 4.5)	6.3 (± 4.5)	6.3 (± 4.5)
Missing	168 (4.4%)	150 (4.3%)	140 (4.2%)	139 (4.1%)
**Maternal EPDS at 32 Weeks Prenatal**				
Mean (± SD)	6.4 (± 4.8)	6.4 (± 4.8)	6.5 (± 4.8)	6.3 (± 4.8)
Missing	134 (3.5%)	122 (3.5%)	108 (3.2%)	112 (3.3%)
**Maternal EPDS at 8 Weeks**				
Mean (± SD)	5.6 (± 4.6)	5.6 (± 4.5)	5.7 (± 4.6)	5.6 (± 4.5)
Missing	196 (5.2%)	190 (5.4%)	161 (4.8%)	160 (4.7%)
**Maternal EPDS at 8 Months**				
Mean (± SD)	5.1 (± 4.5)	5.0 (± 4.5)	5.1 (± 4.6)	5.0 (± 4.5)
Missing	213 (5.6%)	196 (5.6%)	193 (5.8%)	184 (5.4%)
**Maternal EPDS at 21 Months**				
Mean (± SD)	5.3 (± 4.6)	5.4 (± 4.5)	5.4 (± 4.6)	5.3 (± 4.5)
Missing	348 (9.2%)	321 (9.2%)	307 (9.2%)	255 (7.5%)
**Maternal EPDS at 33 Months**				
Mean (± SD)	5.8 (± 4.8)	5.9 (± 4.9)	5.9 (± 4.8)	5.8 (± 4.8)
Missing	458 (12.1%)	386 (11.0%)	386 (11.6%)	357 (10.5%)
**Maternal EPDS at 5 Years**				
Mean (± SD)	5.6 (± 4.7)	5.7 (± 4.7)	5.7 (± 4.8)	5.7 (± 4.8)
Missing	525 (13.8%)	492 (14.0%)	437 (13.1%)	426 (12.6%)
**Maternal EPDS at 6 Years**				
Mean (± SD)	6.0 (± 5.0)	6.0 (± 5.0)	6.0 (± 5.0)	6.0 (± 5.0)
Missing	567 (14.9%)	519 (14.8%)	467 (14.0%)	455 (13.4%)
**Maternal EPDS at 8 Years**				
Mean (± SD)	5.8 (± 5.0)	5.8 (± 5.0)	5.8 (± 5.0)	5.8 (± 5.0)
Missing	747 (19.7%)	676 (19.3%)	610 (18.3%)	582 (17.2%)
**Maternal EPDS at 11 Years**				
Mean (± SD)	5.5 (± 5.2)	5.5 (± 5.1)	5.6 (± 5.2)	5.5 (± 5.1)
Missing	725 (19.1%)	643 (18.3%)	571 (17.1%)	566 (16.7%)

© 2026 Feibel A et al. *JAMA Network Open*.

	Dep Sx (EPDS) (N=3795)	Anx Sx (SCAARED) (N=3505)	Psych Exp (PLIKS) (N=3342)	AUD Sx (AUDIT) (N=3392)
**Maternal EPDS at 18 Years**				
Mean (± SD)	7.1 (± 5.3)	7.2 (± 5.2)	7.1 (± 5.1)	7.2 (± 5.3)
Missing	1627 (42.9%)	1455 (41.5%)	1296 (38.8%)	1275 (37.6%)
**Maternal EPDS at 21 Years**				
Mean (± SD)	6.6 (± 5.7)	6.7 (± 5.7)	6.7 (± 5.6)	6.6 (± 5.7)
Missing	1462 (38.5%)	1310 (37.4%)	1148 (34.4%)	1107 (32.6%)

SD = standard deviation, Dep = Depression, Anx = Anxiety, SCAARED = Screen for Adult Anxiety Related Disorders, PEs = psychotic experiences (determined in semi-structured interviews), and AUDIT = Alcohol Use Disorders Identification Test.

© 2026 Feibel A et al. *JAMA Network Open*.

eTable 2. Descriptive Statistics for Paternal Edinburgh Postnatal Depression Scale (EPDS) Scores (0-30) From 18 Weeks' Gestation to 21 Years, Stratified by Outcome Variables

	Dep Sx (EPDS) (N=3795)	Anx Sx (SCAARED) (N=3505)	Psych Exp (PLIKS) (N=3342)	AUD Sx (AUDIT) (N=3392)
**Paternal EPDS at 18 Weeks Prenatal**				
Mean (± SD)	3.9 (± 3.7)	3.9 (± 3.8)	3.9 (± 3.7)	3.9 (± 3.7)
Missing	703 (18.5%)	663 (18.9%)	584 (17.5%)	609 (18.0%)
**Paternal EPDS at 8 Weeks**				
Mean (± SD)	3.6 (± 3.7)	3.6 (± 3.7)	3.7 (± 3.7)	3.6 (± 3.6)
Missing	952 (25.1%)	890 (25.4%)	824 (24.7%)	818 (24.1%)
**Paternal EPDS at 8 Months**				
Mean (± SD)	3.2 (± 3.5)	3.2 (± 3.6)	3.2 (± 3.5)	3.2 (± 3.5)
Missing	1214 (32.0%)	1107 (31.6%)	1038 (31.1%)	1033 (30.5%)
**Paternal EPDS at 21 Months**				
Mean (± SD)	3.5 (± 3.7)	3.4 (± 3.6)	3.4 (± 3.7)	3.4 (± 3.7)
Missing	1470 (38.7%)	1321 (37.7%)	1242 (37.2%)	1217 (35.9%)
**Paternal EPDS at 33 Months**				
Mean (± SD)	3.6 (± 3.8)	3.6 (± 3.8)	3.7 (± 3.8)	3.7 (± 3.8)
Missing	1575 (41.5%)	1453 (41.5%)	1368 (40.9%)	1344 (39.6%)
**Paternal EPDS at 5 Years**				
Mean (± SD)	3.8 (± 3.8)	3.9 (± 3.9)	3.8 (± 3.9)	3.8 (± 3.8)
Missing	1862 (49.1%)	1687 (48.1%)	1571 (47.0%)	1595 (47.0%)
**Paternal EPDS at 6 Years**				
Mean (± SD)	4.4 (± 4.3)	4.4 (± 4.3)	4.4 (± 4.3)	4.4 (± 4.3)
Missing	1816 (47.9%)	1654 (47.2%)	1539 (46.1%)	1549 (45.7%)
**Paternal EPDS at 8 Years**				
Mean (± SD)	4.1 (± 4.3)	4.1 (± 4.3)	4.2 (± 4.2)	4.1 (± 4.3)
Missing	1977 (52.1%)	1814 (51.8%)	1657 (49.6%)	1679 (49.5%)
**Paternal EPDS at 11 Years**				
Mean (± SD)	3.8 (± 4.3)	3.9 (± 4.3)	3.9 (± 4.3)	3.8 (± 4.3)
Missing	2050 (54.0%)	1883 (53.7%)	1751 (52.4%)	1767 (52.1%)
**Paternal EPDS at 21 Years**				
Mean (± SD)	5.7 (± 4.7)	5.9 (± 4.9)	5.7 (± 4.7)	5.8 (± 4.8)
Missing	2379 (62.7%)	2180 (62.2%)	1955 (58.5%)	2044 (60.3%)

© 2026 Feibel A et al. *JAMA Network Open*.

SD = standard deviation, Dep = Depression, Anx = Anxiety, SCAARED = Screen for Adult Anxiety Related Disorders, PEs = psychotic experiences (determined in semi-structured interviews), and AUDIT = Alcohol Use Disorders Identification Test.

© 2026 Feibel A et al. *JAMA Network Open*.

eTable 3. Regression Table Between Maternal Edinburgh Postnatal Depression Scale (EPDS) and Offspring Age 27 EPDS Scores

Term	Beta	SE	RIV	p value	p < 0.05
PRS Panic Disorder Offspring	0.034	0.037	0.272	0.355	
PRS Bipolar Disorder Offspring	0.006	0.048	0.373	0.908	
PRS Major Depressive Disorder Offspring	0.186	0.046	0.353	0.000	*
PRS Schizophrenia Offspring	0.001	0.049	0.351	0.987	
PRS Alcohol Use Disorder Offspring	0.010	0.041	0.292	0.802	
PRS Anxiety Offspring	0.037	0.044	0.459	0.406	
PRS Panic Disorder Maternal	0.023	0.038	0.308	0.538	
PRS Bipolar Disorder Maternal	-0.029	0.049	0.387	0.555	
PRS Major Depressive Disorder Maternal	0.015	0.044	0.269	0.734	
PRS Schizophrenia Maternal	0.001	0.049	0.362	0.988	
PRS Alcohol Use Disorder Maternal	0.010	0.042	0.344	0.811	
PRS Anxiety Maternal	-0.021	0.042	0.378	0.628	
Parity = 1	-0.002	0.075	0.046	0.981	
Parity >= 2	0.032	0.107	0.063	0.764	
Crowding Index >0.5 - 0.75	0.054	0.077	0.060	0.479	
Crowding Index >0.75 - 1	0.168	0.105	0.071	0.109	
Crowding Index > 1	0.066	0.178	0.054	0.710	
Maternal Social Class = II	0.066	0.129	0.131	0.612	
Maternal Social Class = III (non-manual)	0.042	0.141	0.156	0.764	
Maternal Social Class = III (manual)	-0.213	0.183	0.135	0.245	
Maternal Social Class = IV	0.037	0.176	0.144	0.832	
Maternal Social Class = V	-0.382	0.317	0.041	0.227	
Paternal Social Class = II	0.074	0.098	0.072	0.451	
Paternal Social Class = III (non-manual)	0.051	0.123	0.101	0.677	
Paternal Social Class = III (manual)	0.221	0.115	0.115	0.055	
Paternal Social Class = IV	0.054	0.154	0.124	0.724	
Paternal Social Class = V	0.307	0.238	0.025	0.196	
Maternal Marital Status at Enrollment = 1st Marriage	0.025	0.094	0.043	0.788	
Maternal Marital Status at Enrollment = 2nd or 3rd Marriage	0.031	0.147	0.035	0.832	
Maternal Marital Status at Enrollment = Widowed or Divorced or Separated	-0.207	0.170	0.037	0.222	

© 2026 Feibel A et al. *JAMA Network Open*.

Term	Beta	SE	RIV	p value	p < 0.05
Maternal Age (Years)	-0.007	0.009	0.036	0.461	
Paternal Age (Years)	-0.004	0.007	0.045	0.614	
Maternal Education (Highest Level) = Vocational	-0.309	0.141	0.075	0.028	*
Maternal Education (Highest Level) = O level	-0.127	0.110	0.070	0.249	
Maternal Education (Highest Level) = A level	-0.158	0.121	0.083	0.192	
Maternal Education (Highest Level) = Degree	-0.334	0.144	0.082	0.021	*
Paternal Education (Highest Level) = Vocational	0.056	0.131	0.067	0.667	
Paternal Education (Highest Level) = O level	0.028	0.106	0.121	0.794	
Paternal Education (Highest Level) = A level	0.080	0.100	0.111	0.421	
Paternal Education (Highest Level) = Degree	0.065	0.124	0.106	0.598	
Sex = Female	0.638	0.060	0.007	0.000	*

SE = standard error. RIV = relative increase in variance. The crowding index was calculated as the number of household members per room. Baseline characteristics included sex = male, parity = 0, crowding index < 0.5, maternal and paternal social classes = I, maternal marital status at enrollment = never married, and the highest level of maternal and paternal education = Certificate of Secondary Education (CSE). For parental social class: I = professional occupations, II = managerial and technical occupations, III (non-manual) = skilled non-manual occupations, III (manual) = skilled manual occupations, IV = partly skilled occupations, and V = unskilled occupations. P<0.05 = *.

© 2026 Feibel A et al. *JAMA Network Open*.

eTable 4. Regression Table Between Paternal Edinburgh Postnatal Depression Scale (EPDS) and Offspring Age 27 EPDS Scores

Term	Beta	SE	RIV	p value	p < 0.05
PRS Panic Disorder Offspring	0.032	0.041	0.238	0.434	
PRS Bipolar Disorder Offspring	0.009	0.053	0.399	0.867	
PRS Major Depressive Disorder Offspring	0.196	0.050	0.310	0.000	*
PRS Schizophrenia Offspring	0.000	0.054	0.319	0.999	
PRS Alcohol Use Disorder Offspring	-0.004	0.046	0.305	0.930	
PRS Anxiety Offspring	0.024	0.047	0.392	0.609	
PRS Panic Disorder Maternal	0.026	0.041	0.284	0.530	
PRS Bipolar Disorder Maternal	-0.014	0.054	0.390	0.789	
PRS Major Depressive Disorder Maternal	0.022	0.049	0.273	0.648	
PRS Schizophrenia Maternal	-0.002	0.053	0.318	0.968	
PRS Alcohol Use Disorder Maternal	0.022	0.045	0.305	0.631	
PRS Anxiety Maternal	-0.008	0.046	0.347	0.857	
Parity = 1	0.032	0.081	0.035	0.696	
Parity >= 2	0.084	0.118	0.059	0.477	
Crowding Index >0.5 - 0.75	0.020	0.084	0.062	0.810	
Crowding Index >0.75 - 1	0.143	0.118	0.083	0.225	
Crowding Index > 1	0.094	0.212	0.052	0.657	
Maternal Social Class = II	0.024	0.138	0.123	0.860	
Maternal Social Class = III (non-manual)	0.035	0.152	0.142	0.818	
Maternal Social Class = III (manual)	-0.207	0.198	0.116	0.297	
Maternal Social Class = IV	0.050	0.194	0.158	0.798	
Maternal Social Class = V	-0.174	0.343	0.039	0.612	
Paternal Social Class = II	0.072	0.103	0.057	0.489	
Paternal Social Class = III (non-manual)	0.001	0.132	0.062	0.992	
Paternal Social Class = III (manual)	0.265	0.123	0.091	0.031	*
Paternal Social Class = IV	0.040	0.170	0.112	0.813	
Paternal Social Class = V	0.136	0.270	0.028	0.615	
Maternal Marital Status at Enrollment = 1st Marriage	0.081	0.108	0.052	0.455	
Maternal Marital Status at Enrollment = 2nd or 3rd Marriage	0.238	0.163	0.037	0.145	
Maternal Marital Status at Enrollment = Widowed or Divorced or Separated	-0.078	0.196	0.040	0.689	

© 2026 Feibel A et al. *JAMA Network Open*.

Term	Beta	SE	RIV	p value	p < 0.05
Maternal Age (Years)	-0.007	0.011	0.028	0.521	
Paternal Age (Years)	-0.010	0.008	0.024	0.205	
Maternal Education (Highest Level) = Vocational	-0.373	0.160	0.079	0.019	*
Maternal Education (Highest Level) = O level	-0.165	0.126	0.075	0.193	
Maternal Education (Highest Level) = A level	-0.196	0.138	0.079	0.157	
Maternal Education (Highest Level) = Degree	-0.344	0.161	0.072	0.033	*
Paternal Education (Highest Level) = Vocational	0.179	0.147	0.067	0.222	
Paternal Education (Highest Level) = O level	0.053	0.118	0.087	0.655	
Paternal Education (Highest Level) = A level	0.049	0.112	0.073	0.660	
Paternal Education (Highest Level) = Degree	0.106	0.136	0.071	0.434	
Sex = Female	0.649	0.067	0.011	0.000	*

SE = standard error. RIV = relative increase in variance. The crowding index was calculated as the number of household members per room. Baseline characteristics included sex = male, parity = 0, crowding index < 0.5, maternal and paternal social classes = I, maternal marital status at enrollment = never married, and the highest level of maternal and paternal education = Certificate of Secondary Education (CSE). For parental social class: I = professional occupations, II = managerial and technical occupations, III (non-manual) = skilled non-manual occupations, III (manual) = skilled manual occupations, IV = partly skilled occupations, and V = unskilled occupations. P<0.05 = *.

© 2026 Feibel A et al. *JAMA Network Open*.

eTable 5. Regression Table Between Maternal Edinburgh Postnatal Depression Scale (EPDS) and Offspring Age 25 Screen for Adult Anxiety Related Disorders (SCAARED) Score

Term	Beta	SE	RIV	p value	p < 0.05
PRS Panic Disorder Offspring	0.016	0.039	0.310	0.687	
PRS Bipolar Disorder Offspring	-0.035	0.048	0.234	0.469	
PRS Major Depressive Disorder Offspring	0.221	0.047	0.299	0.000	*
PRS Schizophrenia Offspring	0.038	0.052	0.334	0.462	
PRS Alcohol Use Disorder Offspring	0.034	0.044	0.319	0.435	
PRS Anxiety Offspring	0.025	0.044	0.322	0.572	
PRS Panic Disorder Maternal	0.005	0.040	0.331	0.904	
PRS Bipolar Disorder Maternal	-0.007	0.051	0.420	0.888	
PRS Major Depressive Disorder Maternal	-0.027	0.048	0.354	0.576	
PRS Schizophrenia Maternal	0.005	0.051	0.367	0.924	
PRS Alcohol Use Disorder Maternal	-0.045	0.043	0.281	0.297	
PRS Anxiety Maternal	0.005	0.044	0.346	0.914	
Parity = 1	-0.052	0.078	0.042	0.505	
Parity >= 2	-0.034	0.109	0.055	0.755	
Crowding Index >0.5 - 0.75	0.198	0.080	0.051	0.013	*
Crowding Index >0.75 - 1	0.293	0.109	0.073	0.007	*
Crowding Index > 1	-0.017	0.178	0.048	0.922	
Maternal Social Class = II	0.172	0.131	0.130	0.189	
Maternal Social Class = III (non-manual)	0.101	0.141	0.134	0.476	
Maternal Social Class = III (manual)	0.222	0.191	0.123	0.244	
Maternal Social Class = IV	0.076	0.185	0.180	0.683	
Maternal Social Class = V	-0.035	0.326	0.039	0.914	
Paternal Social Class = II	0.028	0.099	0.065	0.773	
Paternal Social Class = III (non-manual)	-0.057	0.126	0.089	0.652	
Paternal Social Class = III (manual)	-0.033	0.120	0.132	0.784	
Paternal Social Class = IV	0.015	0.155	0.085	0.922	
Paternal Social Class = V	0.275	0.266	0.023	0.302	
Maternal Marital Status at Enrollment = 1st Marriage	-0.148	0.101	0.046	0.141	
Maternal Marital Status at Enrollment = 2nd or 3rd Marriage	-0.224	0.155	0.036	0.150	
Maternal Marital Status at Enrollment = Widowed or Divorced or Separated	-0.002	0.173	0.028	0.990	

© 2026 Feibel A et al. *JAMA Network Open*.

Term	Beta	SE	RIV	p value	p < 0.05
Maternal Age (Years)	0.010	0.010	0.037	0.297	
Paternal Age (Years)	-0.001	0.008	0.048	0.886	
Maternal Education (Highest Level) = Vocational	-0.270	0.149	0.073	0.071	
Maternal Education (Highest Level) = O level	-0.071	0.116	0.074	0.541	
Maternal Education (Highest Level) = A level	-0.138	0.128	0.077	0.278	
Maternal Education (Highest Level) = Degree	-0.310	0.151	0.082	0.040	*
Paternal Education (Highest Level) = Vocational	-0.002	0.139	0.089	0.986	
Paternal Education (Highest Level) = O level	-0.060	0.111	0.109	0.587	
Paternal Education (Highest Level) = A level	0.073	0.106	0.102	0.494	
Paternal Education (Highest Level) = Degree	0.140	0.129	0.101	0.279	
Sex = Female	0.907	0.063	0.008	0.000	*

SE = standard error. RIV = relative increase in variance. The crowding index was calculated as the number of household members per room. Baseline characteristics included sex = male, parity = 0, crowding index < 0.5, maternal and paternal social classes = I, maternal marital status at enrollment = never married, and the highest level of maternal and paternal education = Certificate of Secondary Education (CSE). For parental social class: I = professional occupations, II = managerial and technical occupations, III (non-manual) = skilled non-manual occupations, III (manual) = skilled manual occupations, IV = partly skilled occupations, and V = unskilled occupations. P<0.05 = *.

© 2026 Feibel A et al. *JAMA Network Open*.

eTable 6. Regression Table Between Paternal Edinburgh Postnatal Depression Scale (EPDS) and Offspring Age 25 Screen for Adult Anxiety Related Disorders (SCAARED) Score

Term	Beta	SE	RIV	p value	p < 0.05
PRS Panic Disorder Offspring	0.000	0.044	0.352	0.992	
PRS Bipolar Disorder Offspring	-0.040	0.053	0.258	0.448	
PRS Major Depressive Disorder Offspring	0.215	0.054	0.360	0.000	*
PRS Schizophrenia Offspring	0.052	0.058	0.344	0.364	
PRS Alcohol Use Disorder Offspring	0.024	0.048	0.304	0.616	
PRS Anxiety Offspring	0.017	0.048	0.301	0.726	
PRS Panic Disorder Maternal	0.010	0.044	0.343	0.828	
PRS Bipolar Disorder Maternal	-0.015	0.056	0.407	0.793	
PRS Major Depressive Disorder Maternal	-0.002	0.052	0.335	0.967	
PRS Schizophrenia Maternal	0.012	0.056	0.337	0.829	
PRS Alcohol Use Disorder Maternal	-0.027	0.048	0.304	0.578	
PRS Anxiety Maternal	0.008	0.048	0.340	0.870	
Parity = 1	-0.050	0.086	0.042	0.558	
Parity >= 2	0.042	0.120	0.049	0.728	
Crowding Index >0.5 - 0.75	0.137	0.090	0.061	0.127	
Crowding Index >0.75 - 1	0.250	0.122	0.061	0.041	*
Crowding Index > 1	-0.051	0.212	0.045	0.809	
Maternal Social Class = II	0.077	0.141	0.123	0.585	
Maternal Social Class = III (non-manual)	0.061	0.155	0.132	0.691	
Maternal Social Class = III (manual)	0.221	0.212	0.125	0.298	
Maternal Social Class = IV	0.044	0.204	0.167	0.831	
Maternal Social Class = V	0.074	0.367	0.037	0.839	
Paternal Social Class = II	0.082	0.106	0.058	0.437	
Paternal Social Class = III (non-manual)	-0.029	0.137	0.068	0.830	
Paternal Social Class = III (manual)	0.037	0.129	0.099	0.777	
Paternal Social Class = IV	0.044	0.172	0.073	0.800	
Paternal Social Class = V	-0.057	0.306	0.026	0.851	
Maternal Marital Status at Enrollment = 1st Marriage	-0.112	0.118	0.035	0.341	
Maternal Marital Status at Enrollment = 2nd or 3rd Marriage	-0.134	0.173	0.036	0.438	
Maternal Marital Status at Enrollment = Widowed or Divorced or Separated	-0.007	0.199	0.031	0.971	

© 2026 Feibel A et al. *JAMA Network Open*.

Term	Beta	SE	RIV	p value	p < 0.05
Maternal Age (Years)	0.018	0.011	0.026	0.113	
Paternal Age (Years)	-0.010	0.008	0.026	0.257	
Maternal Education (Highest Level) = Vocational	-0.380	0.171	0.078	0.026	*
Maternal Education (Highest Level) = O level	-0.133	0.134	0.078	0.321	
Maternal Education (Highest Level) = A level	-0.171	0.145	0.066	0.239	
Maternal Education (Highest Level) = Degree	-0.344	0.169	0.059	0.042	*
Paternal Education (Highest Level) = Vocational	0.077	0.157	0.085	0.625	
Paternal Education (Highest Level) = O level	0.060	0.126	0.093	0.634	
Paternal Education (Highest Level) = A level	0.174	0.120	0.080	0.148	
Paternal Education (Highest Level) = Degree	0.213	0.144	0.076	0.137	
Sex = Female	0.880	0.069	0.013	0.000	*

SE = standard error. RIV = relative increase in variance. The crowding index was calculated as the number of household members per room. Baseline characteristics included sex = male, parity = 0, crowding index < 0.5, maternal and paternal social classes = I, maternal marital status at enrollment = never married, and the highest level of maternal and paternal education = Certificate of Secondary Education (CSE). For parental social class: I = professional occupations, II = managerial and technical occupations, III (non-manual) = skilled non-manual occupations, III (manual) = skilled manual occupations, IV = partly skilled occupations, and V = unskilled occupations. P<0.05 = *.

© 2026 Feibel A et al. *JAMA Network Open*.

eTable 7. Regression Table Between Maternal Edinburgh Postnatal Depression Scale (EPDS) and Offspring "Definite" or "Suspected" Psychotic Experiences From 12-24 (Measured at 24 Using the Psychotic-Like Symptoms Interview [PLIKSi] Interview)

Term	Beta	SE	RIV	p value	p < 0.05
Intercept	-3.143	0.629	0.089	0.000	*
PRS Panic Disorder Offspring	-0.055	0.076	0.261	0.474	
PRS Bipolar Disorder Offspring	-0.039	0.094	0.275	0.681	
PRS Major Depressive Disorder Offspring	0.173	0.091	0.304	0.057	
PRS Schizophrenia Offspring	0.040	0.100	0.335	0.690	
PRS Alcohol Use Disorder Offspring	0.053	0.086	0.348	0.534	
PRS Anxiety Offspring	-0.023	0.084	0.252	0.788	
PRS Panic Disorder Maternal	0.050	0.076	0.290	0.515	
PRS Bipolar Disorder Maternal	0.023	0.096	0.317	0.808	
PRS Major Depressive Disorder Maternal	-0.016	0.091	0.276	0.865	
PRS Schizophrenia Maternal	-0.150	0.098	0.338	0.126	
PRS Alcohol Use Disorder Maternal	-0.005	0.087	0.349	0.956	
PRS Anxiety Maternal	0.072	0.084	0.279	0.395	
Parity = 1	-0.087	0.154	0.085	0.574	
Parity >= 2	-0.465	0.221	0.102	0.035	*
Crowding Index >0.5 - 0.75	0.032	0.162	0.087	0.844	
Crowding Index >0.75 - 1	0.400	0.201	0.101	0.046	*
Crowding Index > 1	0.243	0.315	0.046	0.440	
Maternal Social Class = II	0.395	0.291	0.160	0.175	
Maternal Social Class = III (non-manual)	0.624	0.312	0.183	0.046	*
Maternal Social Class = III (manual)	0.593	0.388	0.135	0.127	
Maternal Social Class = IV	0.736	0.373	0.183	0.048	*
Maternal Social Class = V	1.038	0.559	0.053	0.063	
Paternal Social Class = II	0.097	0.207	0.068	0.639	
Paternal Social Class = III (non-manual)	0.003	0.265	0.109	0.990	
Paternal Social Class = III (manual)	0.157	0.239	0.094	0.511	
Paternal Social Class = IV	0.132	0.307	0.095	0.668	
Paternal Social Class = V	0.173	0.476	0.026	0.717	
Maternal Marital Status at Enrollment = 1st Marriage	-0.480	0.171	0.048	0.005	*
Maternal Marital Status at Enrollment = 2nd or 3rd Marriage	-0.185	0.278	0.056	0.505	

© 2026 Feibel A et al. *JAMA Network Open*.

Term	Beta	SE	RIV	p value	p < 0.05
Maternal Marital Status at Enrollment = Widowed or Divorced or Separated	-0.026	0.284	0.044	0.928	
Maternal Age (Years)	-0.009	0.019	0.036	0.632	
Paternal Age (Years)	0.022	0.014	0.047	0.098	
Maternal Education (Highest Level) = Vocational	0.268	0.266	0.055	0.314	
Maternal Education (Highest Level) = O level	0.111	0.218	0.056	0.612	
Maternal Education (Highest Level) = A level	-0.256	0.248	0.049	0.302	
Maternal Education (Highest Level) = Degree	0.193	0.286	0.043	0.500	
Paternal Education (Highest Level) = Vocational	0.113	0.258	0.091	0.661	
Paternal Education (Highest Level) = O level	-0.092	0.211	0.125	0.662	
Paternal Education (Highest Level) = A level	0.035	0.201	0.124	0.861	
Paternal Education (Highest Level) = Degree	0.238	0.249	0.101	0.338	
Sex = Female	-0.172	0.119	0.005	0.148	

SE = standard error. RIV = relative increase in variance. The crowding index was calculated as the number of household members per room. Baseline characteristics included sex = male, parity = 0, crowding index < 0.5, maternal and paternal social classes = I, maternal marital status at enrollment = never married, and the highest level of maternal and paternal education = Certificate of Secondary Education (CSE). For parental social class: I = professional occupations, II = managerial and technical occupations, III (non-manual) = skilled non-manual occupations, III (manual) = skilled manual occupations, IV = partly skilled occupations, and V = unskilled occupations. P<0.05 = *.

© 2026 Feibel A et al. *JAMA Network Open*.

eTable 8. Regression Table Between Paternal Edinburgh Postnatal Depression Scale (EPDS) and Offspring "Definite" or "Suspected" Psychotic Experiences From 12-24 (Measured at 24 Using the Psychotic-Like Symptoms Interview [PLIKSi] Interview)

Term	Beta	SE	RIV	p value	p < 0.05
Intercept	-2.679	0.696	0.070	0.000	*
PRS Panic Disorder Offspring	-0.108	0.085	0.270	0.204	
PRS Bipolar Disorder Offspring	-0.071	0.104	0.256	0.497	
PRS Major Depressive Disorder Offspring	0.186	0.102	0.291	0.069	
PRS Schizophrenia Offspring	0.114	0.111	0.317	0.304	
PRS Alcohol Use Disorder Offspring	0.014	0.094	0.302	0.880	
PRS Anxiety Offspring	-0.003	0.093	0.241	0.977	
PRS Panic Disorder Maternal	0.084	0.085	0.302	0.324	
PRS Bipolar Disorder Maternal	-0.018	0.105	0.262	0.867	
PRS Major Depressive Disorder Maternal	0.003	0.100	0.244	0.977	
PRS Schizophrenia Maternal	-0.147	0.108	0.323	0.176	
PRS Alcohol Use Disorder Maternal	0.022	0.097	0.366	0.821	
PRS Anxiety Maternal	0.066	0.092	0.225	0.471	
Parity = 1	-0.185	0.172	0.069	0.283	
Parity >= 2	-0.398	0.242	0.080	0.100	
Crowding Index >0.5 - 0.75	0.071	0.179	0.073	0.694	
Crowding Index >0.75 - 1	0.463	0.226	0.083	0.040	*
Crowding Index > 1	0.326	0.383	0.037	0.395	
Maternal Social Class = II	0.269	0.314	0.161	0.391	
Maternal Social Class = III (non-manual)	0.643	0.338	0.192	0.057	
Maternal Social Class = III (manual)	0.625	0.425	0.148	0.141	
Maternal Social Class = IV	0.672	0.414	0.191	0.105	
Maternal Social Class = V	0.016	0.823	0.033	0.984	
Paternal Social Class = II	0.243	0.222	0.060	0.274	
Paternal Social Class = III (non-manual)	0.116	0.286	0.071	0.684	
Paternal Social Class = III (manual)	0.294	0.261	0.080	0.259	
Paternal Social Class = IV	-0.051	0.359	0.070	0.887	
Paternal Social Class = V	0.322	0.554	0.019	0.561	
Maternal Marital Status at Enrollment = 1st Marriage	-0.262	0.207	0.045	0.206	
Maternal Marital Status at Enrollment = 2nd or 3rd Marriage	0.026	0.314	0.046	0.934	

© 2026 Feibel A et al. *JAMA Network Open*.

Term	Beta	SE	RIV	p value	p < 0.05
Maternal Marital Status at Enrollment = Widowed or Divorced or Separated	0.089	0.344	0.046	0.795	
Maternal Age (Years)	-0.013	0.021	0.029	0.543	
Paternal Age (Years)	0.017	0.015	0.023	0.270	
Maternal Education (Highest Level) = Vocational	0.130	0.302	0.061	0.666	
Maternal Education (Highest Level) = O level	-0.124	0.251	0.071	0.622	
Maternal Education (Highest Level) = A level	-0.454	0.285	0.067	0.111	
Maternal Education (Highest Level) = Degree	-0.035	0.323	0.057	0.915	
Paternal Education (Highest Level) = Vocational	-0.001	0.300	0.072	0.996	
Paternal Education (Highest Level) = O level	-0.165	0.239	0.108	0.490	
Paternal Education (Highest Level) = A level	0.024	0.226	0.081	0.916	
Paternal Education (Highest Level) = Degree	0.312	0.274	0.069	0.255	
Sex = Female	-0.283	0.131	0.006	0.031	*

SE = standard error. RIV = relative increase in variance. The crowding index was calculated as the number of household members per room. Baseline characteristics included sex = male, parity = 0, crowding index < 0.5, maternal and paternal social classes = I, maternal marital status at enrollment = never married, and the highest level of maternal and paternal education = Certificate of Secondary Education (CSE). For parental social class: I = professional occupations, II = managerial and technical occupations, III (non-manual) = skilled non-manual occupations, III (manual) = skilled manual occupations, IV = partly skilled occupations, and V = unskilled occupations. P<0.05 = *.

© 2026 Feibel A et al. *JAMA Network Open*.

eTable 9. Regression Table Between Maternal Edinburgh Postnatal Depression Scale (EPDS) and Adult Offspring Alcohol Use Disorder Identification Test (AUDIT) Score

Term	Beta	SE	RIV	p value	p < 0.05
PRS Panic Disorder Offspring	-0.005	0.039	0.303	0.899	
PRS Bipolar Disorder Offspring	-0.015	0.050	0.346	0.772	
PRS Major Depressive Disorder Offspring	-0.051	0.050	0.402	0.301	
PRS Schizophrenia Offspring	0.027	0.053	0.393	0.606	
PRS Alcohol Use Disorder Offspring	0.138	0.044	0.338	0.002	*
PRS Anxiety Offspring	-0.045	0.045	0.350	0.315	
PRS Panic Disorder Maternal	0.025	0.041	0.370	0.534	
PRS Bipolar Disorder Maternal	-0.010	0.051	0.337	0.840	
PRS Major Depressive Disorder Maternal	0.006	0.049	0.394	0.910	
PRS Schizophrenia Maternal	0.012	0.053	0.374	0.825	
PRS Alcohol Use Disorder Maternal	0.029	0.044	0.317	0.510	
PRS Anxiety Maternal	-0.030	0.045	0.335	0.508	
Parity = 1	0.148	0.079	0.038	0.061	
Parity >= 2	0.217	0.113	0.065	0.056	
Crowding Index >0.5 - 0.75	-0.135	0.082	0.053	0.098	
Crowding Index >0.75 - 1	-0.318	0.114	0.085	0.005	*
Crowding Index > 1	-0.473	0.195	0.067	0.015	*
Maternal Social Class = II	-0.056	0.130	0.144	0.665	
Maternal Social Class = III (non-manual)	-0.104	0.141	0.145	0.461	
Maternal Social Class = III (manual)	-0.053	0.189	0.114	0.781	
Maternal Social Class = IV	-0.108	0.186	0.169	0.561	
Maternal Social Class = V	-0.712	0.353	0.037	0.044	*
Paternal Social Class = II	0.096	0.098	0.050	0.329	
Paternal Social Class = III (non-manual)	0.008	0.127	0.074	0.947	
Paternal Social Class = III (manual)	-0.007	0.118	0.069	0.953	
Paternal Social Class = IV	0.107	0.156	0.073	0.491	
Paternal Social Class = V	0.372	0.270	0.016	0.168	
Maternal Marital Status at Enrollment = 1st Marriage	-0.351	0.105	0.038	0.001	*
Maternal Marital Status at Enrollment = 2nd or 3rd Marriage	-0.617	0.161	0.048	0.000	*
Maternal Marital Status at Enrollment = Widowed or Divorced or Separated	-0.371	0.179	0.040	0.038	*

© 2026 Feibel A et al. *JAMA Network Open*.

Term	Beta	SE	RIV	p value	p < 0.05
Maternal Age (Years)	0.023	0.010	0.030	0.021	*
Paternal Age (Years)	-0.009	0.008	0.036	0.222	
Maternal Education (Highest Level) = Vocational	0.060	0.157	0.058	0.703	
Maternal Education (Highest Level) = O level	0.245	0.122	0.060	0.044	*
Maternal Education (Highest Level) = A level	0.314	0.132	0.069	0.018	*
Maternal Education (Highest Level) = Degree	0.380	0.154	0.063	0.014	*
Paternal Education (Highest Level) = Vocational	-0.042	0.150	0.070	0.777	
Paternal Education (Highest Level) = O level	-0.097	0.112	0.085	0.383	
Paternal Education (Highest Level) = A level	0.062	0.106	0.073	0.558	
Paternal Education (Highest Level) = Degree	0.145	0.129	0.073	0.260	
Sex = Female	-0.429	0.064	0.006	0.000	*

SE = standard error. RIV = relative increase in variance. The crowding index was calculated as the number of household members per room. Baseline characteristics included sex = male, parity = 0, crowding index < 0.5, maternal and paternal social classes = I, maternal marital status at enrollment = never married, and the highest level of maternal and paternal education = Certificate of Secondary Education (CSE). For parental social class: I = professional occupations, II = managerial and technical occupations, III (non-manual) = skilled non-manual occupations, III (manual) = skilled manual occupations, IV = partly skilled occupations, and V = unskilled occupations. P<0.05 = *.

© 2026 Feibel A et al. *JAMA Network Open*.

eTable 10. Regression Table Between Paternal Edinburgh Postnatal Depression Scale (EPDS) and Adult Offspring Alcohol Use Disorder Identification Test (AUDIT) Score

Term	Beta	SE	RIV	p value	p < 0.05
PRS Panic Disorder Offspring	0.001	0.043	0.285	0.990	
PRS Bipolar Disorder Offspring	-0.031	0.054	0.299	0.564	
PRS Major Depressive Disorder Offspring	-0.066	0.054	0.374	0.223	
PRS Schizophrenia Offspring	0.046	0.058	0.377	0.426	
PRS Alcohol Use Disorder Offspring	0.141	0.048	0.314	0.004	*
PRS Anxiety Offspring	-0.050	0.049	0.330	0.310	
PRS Panic Disorder Maternal	0.043	0.044	0.348	0.329	
PRS Bipolar Disorder Maternal	0.004	0.055	0.314	0.941	
PRS Major Depressive Disorder Maternal	0.041	0.054	0.376	0.447	
PRS Schizophrenia Maternal	-0.004	0.058	0.382	0.949	
PRS Alcohol Use Disorder Maternal	0.048	0.049	0.333	0.321	
PRS Anxiety Maternal	-0.063	0.049	0.339	0.203	
Parity = 1	0.131	0.088	0.045	0.137	
Parity >= 2	0.225	0.126	0.066	0.074	
Crowding Index >0.5 - 0.75	-0.170	0.090	0.050	0.059	
Crowding Index >0.75 - 1	-0.416	0.128	0.087	0.001	*
Crowding Index > 1	-0.472	0.228	0.077	0.039	*
Maternal Social Class = II	-0.118	0.139	0.138	0.398	
Maternal Social Class = III (non-manual)	-0.129	0.153	0.147	0.399	
Maternal Social Class = III (manual)	-0.032	0.208	0.111	0.876	
Maternal Social Class = IV	-0.060	0.202	0.159	0.766	
Maternal Social Class = V	-0.668	0.384	0.028	0.082	
Paternal Social Class = II	0.147	0.105	0.043	0.162	
Paternal Social Class = III (non-manual)	0.045	0.137	0.061	0.741	
Paternal Social Class = III (manual)	0.073	0.127	0.048	0.568	
Paternal Social Class = IV	0.248	0.173	0.062	0.150	
Paternal Social Class = V	0.545	0.308	0.017	0.077	
Maternal Marital Status at Enrollment = 1st Marriage	-0.281	0.121	0.034	0.021	*
Maternal Marital Status at Enrollment = 2nd or 3rd Marriage	-0.471	0.180	0.042	0.009	*
Maternal Marital Status at Enrollment = Widowed or Divorced or Separated	-0.140	0.204	0.031	0.493	

© 2026 Feibel A et al. *JAMA Network Open*.

Term	Beta	SE	RIV	p value	p < 0.05
Maternal Age (Years)	0.019	0.011	0.018	0.100	
Paternal Age (Years)	-0.011	0.008	0.015	0.209	
Maternal Education (Highest Level) = Vocational	0.054	0.176	0.048	0.760	
Maternal Education (Highest Level) = O level	0.200	0.139	0.054	0.149	
Maternal Education (Highest Level) = A level	0.274	0.150	0.059	0.067	
Maternal Education (Highest Level) = Degree	0.385	0.172	0.062	0.025	*
Paternal Education (Highest Level) = Vocational	-0.053	0.168	0.058	0.751	
Paternal Education (Highest Level) = O level	-0.108	0.124	0.059	0.385	
Paternal Education (Highest Level) = A level	0.093	0.120	0.056	0.441	
Paternal Education (Highest Level) = Degree	0.189	0.142	0.053	0.183	
Sex = Female	-0.401	0.070	0.007	0.000	*

SE = standard error. RIV = relative increase in variance. The crowding index was calculated as the number of household members per room. Baseline characteristics included sex = male, parity = 0, crowding index < 0.5, maternal and paternal social classes = I, maternal marital status at enrollment = never married, and the highest level of maternal and paternal education = Certificate of Secondary Education (CSE). For parental social class: I = professional occupations, II = managerial and technical occupations, III (non-manual) = skilled non-manual occupations, III (manual) = skilled manual occupations, IV = partly skilled occupations, and V = unskilled occupations. P<0.05 = *.

© 2026 Feibel A et al. *JAMA Network Open*.

eTable 11. Chi-Square Tests for Goodness of Fit Between the Distributed Lag Interaction Model (DLIM) (With Sex as the Modifying Variable) and the Distributed Lag Model (DLM) for Each Parental Mental Health Exposure—Offspring Mental Health Outcome Pair

Exposure	Outcome	Sex D2 p value	Sex D2 FDR
Paternal CCEI	AUDIT	0.037	0.482
Maternal CCEI	AUDIT	0.060	0.482
Paternal EPDS	AUDIT	0.102	0.543
Maternal EPDS	PEs	0.320	0.833
Paternal EPDS	PEs	0.333	0.833
Maternal EPDS	AUDIT	0.378	0.833
Paternal CCEI	PEs	0.492	0.833
Maternal CCEI	PEs	0.526	0.833
Paternal CCEI	SCAARED	0.549	0.833
Paternal EPDS	EPDS	0.623	0.833
Maternal EPDS	EPDS	0.638	0.833
Maternal CCEI	SCAARED	0.669	0.833
Maternal CCEI	EPDS	0.677	0.833
Paternal EPDS	SCAARED	0.831	0.923
Maternal EPDS	SCAARED	0.881	0.923
Paternal CCEI	EPDS	0.923	0.923

DLM is the base or null model. FDR = False Discovery Rate. EPDS = Edinburgh Postnatal Depression Scale, CCEI = Crown Crisp Experiential Index anxiety subscore, SCAARED = Screen for Adult Anxiety Related Disorders, PEs = psychotic experiences (determined in semi-structured interviews), and AUDIT = Alcohol Use Disorders Identification Test. P<0.05 = *

© 2026 Feibel A et al. *JAMA Network Open*.

eTable 12. Chi-Square Tests for Goodness of Fit Between the Distributed Lag Interaction Model (DLIM) (With PRS as the Modifying Variable) and the Distributed Lag Model (DLM) for Each Parental Mental Health Exposure—Offspring Mental Health Outcome Pair

Exposure	Outcome	PRS D2 p value	PRS D2 FDR
Maternal CCEI	SCAARED	0.147	0.988
Maternal EPDS	SCAARED	0.189	0.988
Maternal CCEI	EPDS	0.298	0.988
Maternal EPDS	EPDS	0.365	0.988
Maternal EPDS	PEs	0.391	0.988
Paternal EPDS	SCAARED	0.448	0.988
Paternal CCEI	SCAARED	0.516	0.988
Paternal EPDS	AUDIT	0.675	0.988
Paternal CCEI	PEs	0.799	0.988
Maternal CCEI	PEs	0.837	0.988
Maternal EPDS	AUDIT	0.853	0.988
Paternal EPDS	EPDS	0.868	0.988
Paternal EPDS	PEs	0.933	0.988
Maternal CCEI	AUDIT	0.935	0.988
Paternal CCEI	AUDIT	0.945	0.988
Paternal CCEI	EPDS	0.988	0.988

DLM is the base or null model. FDR = False Discovery Rate. EPDS = Edinburgh Postnatal Depression Scale, CCEI = Crown Crisp Experiential Index anxiety subscore, SCAARED = Screen for Adult Anxiety Related Disorders, PEs = psychotic experiences (determined in semi-structured interviews), and AUDIT = Alcohol Use Disorders Identification Test. P<0.05 = *

eTable 13. Descriptive Statistics for Maternal Crown-Crisp Experiential Index (CCEI) Anxiety Subscores (0-16) From 18 Weeks' Gestation to 6 Years, Stratified by Outcome Variables

	Dep Sx (EPDS) (N=3795)	Anx Sx (SCAARED) (N=3505)	Psych Exp (PLIKS) (N=3342)	AUD Sx (AUDIT) (N=3392)
**Maternal CCEI at 18 Weeks Prenatal**				
Mean (± SD)	4.6 (± 3.4)	4.6 (± 3.4)	4.6 (± 3.4)	4.6 (± 3.4)
Missing	204 (5.4%)	180 (5.1%)	175 (5.2%)	172 (5.1%)
**Maternal CCEI at 32 Weeks Prenatal**				
Mean (± SD)	4.8 (± 3.5)	4.8 (± 3.5)	4.8 (± 3.5)	4.7 (± 3.4)
Missing	170 (4.5%)	150 (4.3%)	139 (4.2%)	139 (4.1%)
**Maternal CCEI at 8 Weeks**				
Mean (± SD)	3.2 (± 3.2)	3.2 (± 3.1)	3.3 (± 3.2)	3.2 (± 3.2)
Missing	190 (5.0%)	188 (5.4%)	162 (4.8%)	158 (4.7%)
**Maternal CCEI at 8 Months**				
Mean (± SD)	3.4 (± 3.2)	3.4 (± 3.2)	3.5 (± 3.2)	3.4 (± 3.2)
Missing	213 (5.6%)	196 (5.6%)	194 (5.8%)	184 (5.4%)
**Maternal CCEI at 21 Months**				
Mean (± SD)	3.6 (± 3.2)	3.6 (± 3.2)	3.6 (± 3.2)	3.6 (± 3.2)
Missing	348 (9.2%)	321 (9.2%)	309 (9.2%)	255 (7.5%)
**Maternal CCEI at 33 Months**				
Mean (± SD)	4.5 (± 3.3)	4.4 (± 3.3)	4.5 (± 3.4)	4.4 (± 3.3)
Missing	454 (12.0%)	389 (11.1%)	384 (11.5%)	350 (10.3%)
**Maternal CCEI at 5 Years**				
Mean (± SD)	4.3 (± 3.3)	4.3 (± 3.3)	4.4 (± 3.3)	4.3 (± 3.3)
Missing	524 (13.8%)	494 (14.1%)	434 (13.0%)	427 (12.6%)
**Maternal CCEI at 6 Years**				
Mean (± SD)	5.0 (± 3.5)	5.0 (± 3.4)	5.0 (± 3.5)	4.9 (± 3.4)
Missing	570 (15.0%)	521 (14.9%)	469 (14.0%)	456 (13.4%)

SD = standard deviation, Dep = Depression, Anx = Anxiety, SCAARED = Screen for Adult Anxiety Related Disorders, PEs = psychotic experiences (determined in semi-structured interviews), and AUDIT = Alcohol Use Disorders Identification Test.

© 2026 Feibel A et al. *JAMA Network Open*.

eTable 14. Descriptive Statistics for Paternal Crown-Crisp Experiential Index (CCEI) Anxiety Subscores (0-16) From 18 Weeks' Gestation to 6 Years, Stratified by Outcome Variables

	Dep Sx (EPDS) (N=3795)	Anx Sx (SCAARED) (N=3505)	Psych Exp (PLIKS) (N=3342)	AUD Sx (AUDIT) (N=3392)
**Paternal CCEI at 18 Weeks Prenatal**				
Mean (± SD)	3.0 (± 2.7)	3.0 (± 2.7)	3.0 (± 2.7)	3.0 (± 2.7)
Missing	709 (18.7%)	667 (19.0%)	587 (17.6%)	616 (18.2%)
**Paternal CCEI at 8 Weeks**				
Mean (± SD)	2.5 (± 2.5)	2.5 (± 2.5)	2.6 (± 2.6)	2.5 (± 2.6)
Missing	950 (25.0%)	890 (25.4%)	823 (24.6%)	820 (24.2%)
**Paternal CCEI at 8 Months**				
Mean (± SD)	2.2 (± 2.3)	2.2 (± 2.4)	2.2 (± 2.4)	2.2 (± 2.4)
Missing	1217 (32.1%)	1112 (31.7%)	1043 (31.2%)	1037 (30.6%)
**Paternal CCEI at 21 Months**				
Mean (± SD)	2.5 (± 2.5)	2.5 (± 2.5)	2.5 (± 2.5)	2.5 (± 2.5)
Missing	1471 (38.8%)	1321 (37.7%)	1246 (37.3%)	1219 (35.9%)
**Paternal CCEI at 33 Months**				
Mean (± SD)	3.1 (± 2.6)	3.1 (± 2.7)	3.1 (± 2.6)	3.1 (± 2.6)
Missing	1564 (41.2%)	1442 (41.1%)	1357 (40.6%)	1334 (39.3%)
**Paternal CCEI at 5 Years**				
Mean (± SD)	3.0 (± 2.7)	3.0 (± 2.7)	3.0 (± 2.7)	3.0 (± 2.6)
Missing	1858 (49.0%)	1682 (48.0%)	1571 (47.0%)	1598 (47.1%)
**Paternal CCEI at 6 Years**				
Mean (± SD)	3.7 (± 3.1)	3.6 (± 3.0)	3.7 (± 3.0)	3.7 (± 3.0)
Missing	1819 (47.9%)	1657 (47.3%)	1540 (46.1%)	1549 (45.7%)

SD = standard deviation, Dep = Depression, Anx = Anxiety, SCAARED = Screen for Adult Anxiety Related Disorders, PEs = psychotic experiences (determined in semi-structured interviews), and AUDIT = Alcohol Use Disorders Identification Test.

© 2026 Feibel A et al. *JAMA Network Open*.

eTable 15. Regression Table Between Maternal Crown-Crisp Experiential Index (CCEI) Anxiety Subscores and Offspring Age 27 EPDS Scores

Term	Beta	SE	RIV	p value	p < 0.05
PRS Panic Disorder Offspring	0.035	0.037	0.262	0.352	
PRS Bipolar Disorder Offspring	0.008	0.048	0.384	0.872	
PRS Major Depressive Disorder Offspring	0.187	0.046	0.355	0.000	*
PRS Schizophrenia Offspring	0.000	0.049	0.356	0.993	
PRS Alcohol Use Disorder Offspring	0.014	0.042	0.297	0.739	
PRS Anxiety Offspring	0.034	0.044	0.464	0.446	
PRS Panic Disorder Maternal	0.025	0.038	0.300	0.516	
PRS Bipolar Disorder Maternal	-0.031	0.049	0.394	0.529	
PRS Major Depressive Disorder Maternal	0.020	0.044	0.270	0.647	
PRS Schizophrenia Maternal	0.003	0.049	0.369	0.958	
PRS Alcohol Use Disorder Maternal	0.010	0.042	0.344	0.805	
PRS Anxiety Maternal	-0.021	0.042	0.376	0.614	
Parity = 1	-0.001	0.075	0.048	0.994	
Parity >= 2	0.033	0.107	0.064	0.757	
Crowding Index >0.5 - 0.75	0.059	0.077	0.060	0.445	
Crowding Index >0.75 - 1	0.183	0.105	0.071	0.082	
Crowding Index > 1	0.074	0.179	0.057	0.680	
Maternal Social Class = II	0.068	0.129	0.132	0.597	
Maternal Social Class = III (non-manual)	0.047	0.141	0.152	0.740	
Maternal Social Class = III (manual)	-0.199	0.182	0.131	0.277	
Maternal Social Class = IV	0.046	0.176	0.142	0.795	
Maternal Social Class = V	-0.335	0.316	0.040	0.288	
Paternal Social Class = II	0.077	0.098	0.073	0.432	
Paternal Social Class = III (non-manual)	0.057	0.124	0.102	0.642	
Paternal Social Class = III (manual)	0.222	0.115	0.112	0.053	
Paternal Social Class = IV	0.045	0.154	0.133	0.772	
Paternal Social Class = V	0.291	0.237	0.025	0.220	
Maternal Marital Status at Enrollment = 1st Marriage	0.029	0.094	0.044	0.756	
Maternal Marital Status at Enrollment = 2nd or 3rd Marriage	0.048	0.147	0.033	0.745	
Maternal Marital Status at Enrollment = Widowed or Divorced or Separated	-0.200	0.170	0.037	0.239	

© 2026 Feibel A et al. *JAMA Network Open*.

Term	Beta	SE	RIV	p value	p < 0.05
Maternal Age (Years)	-0.005	0.009	0.033	0.577	
Paternal Age (Years)	-0.003	0.007	0.041	0.628	
Maternal Education (Highest Level) = Vocational	-0.319	0.141	0.074	0.024	*
Maternal Education (Highest Level) = O level	-0.142	0.110	0.070	0.197	
Maternal Education (Highest Level) = A level	-0.182	0.121	0.081	0.134	
Maternal Education (Highest Level) = Degree	-0.369	0.144	0.080	0.011	*
Paternal Education (Highest Level) = Vocational	0.058	0.131	0.067	0.661	
Paternal Education (Highest Level) = O level	0.015	0.106	0.119	0.886	
Paternal Education (Highest Level) = A level	0.060	0.100	0.108	0.548	
Paternal Education (Highest Level) = Degree	0.052	0.124	0.107	0.677	
Sex = Female	0.636	0.060	0.007	0.000	*

SE = standard error. RIV = relative increase in variance. The crowding index was calculated as the number of household members per room. Baseline characteristics included sex = male, parity = 0, crowding index < 0.5, maternal and paternal social classes = I, maternal marital status at enrollment = never married, and the highest level of maternal and paternal education = Certificate of Secondary Education (CSE). For parental social class: I = professional occupations, II = managerial and technical occupations, III (non-manual) = skilled non-manual occupations, III (manual) = skilled manual occupations, IV = partly skilled occupations, and V = unskilled occupations. P<0.05 = *.

© 2026 Feibel A et al. *JAMA Network Open*.

eTable 16. Regression Table Between Paternal Crown-Crisp Experiential Index (CCEI) Anxiety Subscores and Offspring Age 27 EPDS Scores

Term	Beta	SE	RIV	p value	p < 0.05
PRS Panic Disorder Offspring	0.039	0.041	0.246	0.346	
PRS Bipolar Disorder Offspring	0.007	0.054	0.407	0.895	
PRS Major Depressive Disorder Offspring	0.198	0.050	0.306	0.000	*
PRS Schizophrenia Offspring	0.000	0.055	0.329	1.000	
PRS Alcohol Use Disorder Offspring	-0.002	0.046	0.303	0.965	
PRS Anxiety Offspring	0.023	0.048	0.407	0.636	
PRS Panic Disorder Maternal	0.025	0.042	0.292	0.558	
PRS Bipolar Disorder Maternal	-0.012	0.054	0.379	0.821	
PRS Major Depressive Disorder Maternal	0.030	0.049	0.279	0.539	
PRS Schizophrenia Maternal	0.003	0.053	0.334	0.960	
PRS Alcohol Use Disorder Maternal	0.020	0.045	0.300	0.653	
PRS Anxiety Maternal	-0.014	0.047	0.348	0.767	
Parity = 1	0.034	0.082	0.036	0.676	
Parity >= 2	0.081	0.118	0.060	0.492	
Crowding Index >0.5 - 0.75	0.025	0.085	0.058	0.772	
Crowding Index >0.75 - 1	0.144	0.119	0.087	0.227	
Crowding Index > 1	0.107	0.215	0.055	0.620	
Maternal Social Class = II	0.058	0.139	0.123	0.679	
Maternal Social Class = III (non-manual)	0.062	0.152	0.138	0.683	
Maternal Social Class = III (manual)	-0.171	0.199	0.111	0.389	
Maternal Social Class = IV	0.047	0.198	0.171	0.814	
Maternal Social Class = V	-0.120	0.352	0.040	0.733	
Paternal Social Class = II	0.064	0.104	0.055	0.539	
Paternal Social Class = III (non-manual)	-0.007	0.133	0.065	0.958	
Paternal Social Class = III (manual)	0.257	0.124	0.082	0.038	*
Paternal Social Class = IV	0.032	0.171	0.100	0.853	
Paternal Social Class = V	0.152	0.275	0.021	0.580	
Maternal Marital Status at Enrollment = 1st Marriage	0.060	0.109	0.049	0.583	
Maternal Marital Status at Enrollment = 2nd or 3rd Marriage	0.219	0.165	0.032	0.182	
Maternal Marital Status at Enrollment = Widowed or Divorced or Separated	-0.092	0.199	0.039	0.642	

© 2026 Feibel A et al. *JAMA Network Open*.

Term	Beta	SE	RIV	p value	p < 0.05
Maternal Age (Years)	-0.009	0.011	0.024	0.374	
Paternal Age (Years)	-0.010	0.008	0.021	0.221	
Maternal Education (Highest Level) = Vocational	-0.370	0.162	0.076	0.022	*
Maternal Education (Highest Level) = O level	-0.187	0.128	0.068	0.142	
Maternal Education (Highest Level) = A level	-0.228	0.139	0.070	0.102	
Maternal Education (Highest Level) = Degree	-0.357	0.162	0.063	0.027	*
Paternal Education (Highest Level) = Vocational	0.164	0.148	0.054	0.270	
Paternal Education (Highest Level) = O level	0.057	0.119	0.079	0.633	
Paternal Education (Highest Level) = A level	0.042	0.112	0.058	0.707	
Paternal Education (Highest Level) = Degree	0.090	0.136	0.062	0.509	
Sex = Female	0.670	0.067	0.007	0.000	*

SE = standard error. RIV = relative increase in variance. The crowding index was calculated as the number of household members per room. Baseline characteristics included sex = male, parity = 0, crowding index < 0.5, maternal and paternal social classes = I, maternal marital status at enrollment = never married, and the highest level of maternal and paternal education = Certificate of Secondary Education (CSE). For parental social class: I = professional occupations, II = managerial and technical occupations, III (non-manual) = skilled non-manual occupations, III (manual) = skilled manual occupations, IV = partly skilled occupations, and V = unskilled occupations. P<0.05 = *.

© 2026 Feibel A et al. *JAMA Network Open*.

eTable 17. Regression Table Between Maternal Crown-Crisp Experiential Index (CCEI) Anxiety Subscores and Offspring Age 25 Screen for Adult Anxiety Related Disorders (SCAARED) Score

Term	Beta	SE	RIV	p value	p < 0.05
PRS Panic Disorder Offspring	0.018	0.039	0.313	0.652	
PRS Bipolar Disorder Offspring	-0.028	0.048	0.238	0.562	
PRS Major Depressive Disorder Offspring	0.218	0.047	0.305	0.000	*
PRS Schizophrenia Offspring	0.035	0.052	0.336	0.493	
PRS Alcohol Use Disorder Offspring	0.040	0.043	0.307	0.361	
PRS Anxiety Offspring	0.021	0.044	0.325	0.625	
PRS Panic Disorder Maternal	0.004	0.040	0.333	0.921	
PRS Bipolar Disorder Maternal	-0.013	0.051	0.427	0.805	
PRS Major Depressive Disorder Maternal	-0.027	0.048	0.349	0.573	
PRS Schizophrenia Maternal	0.008	0.051	0.373	0.869	
PRS Alcohol Use Disorder Maternal	-0.045	0.043	0.279	0.291	
PRS Anxiety Maternal	0.006	0.044	0.350	0.896	
Parity = 1	-0.062	0.078	0.044	0.423	
Parity >= 2	-0.040	0.109	0.057	0.710	
Crowding Index >0.5 - 0.75	0.204	0.080	0.050	0.011	*
Crowding Index >0.75 - 1	0.299	0.109	0.075	0.006	*
Crowding Index > 1	-0.046	0.177	0.047	0.794	
Maternal Social Class = II	0.185	0.131	0.130	0.156	
Maternal Social Class = III (non-manual)	0.105	0.141	0.131	0.456	
Maternal Social Class = III (manual)	0.246	0.191	0.123	0.197	
Maternal Social Class = IV	0.086	0.185	0.171	0.643	
Maternal Social Class = V	-0.002	0.326	0.039	0.995	
Paternal Social Class = II	0.029	0.098	0.063	0.765	
Paternal Social Class = III (non-manual)	-0.059	0.126	0.091	0.640	
Paternal Social Class = III (manual)	-0.034	0.120	0.130	0.774	
Paternal Social Class = IV	0.015	0.155	0.084	0.921	
Paternal Social Class = V	0.263	0.265	0.022	0.321	
Maternal Marital Status at Enrollment = 1st Marriage	-0.135	0.101	0.049	0.182	
Maternal Marital Status at Enrollment = 2nd or 3rd Marriage	-0.212	0.155	0.037	0.172	
Maternal Marital Status at Enrollment = Widowed or Divorced or Separated	0.000	0.173	0.027	0.999	

© 2026 Feibel A et al. *JAMA Network Open*.

Term	Beta	SE	RIV	p value	p < 0.05
Maternal Age (Years)	0.010	0.010	0.033	0.304	
Paternal Age (Years)	0.000	0.008	0.047	0.997	
Maternal Education (Highest Level) = Vocational	-0.293	0.149	0.068	0.050	*
Maternal Education (Highest Level) = O level	-0.080	0.116	0.064	0.489	
Maternal Education (Highest Level) = A level	-0.157	0.127	0.071	0.217	
Maternal Education (Highest Level) = Degree	-0.352	0.151	0.074	0.020	*
Paternal Education (Highest Level) = Vocational	0.012	0.139	0.089	0.934	
Paternal Education (Highest Level) = O level	-0.068	0.111	0.105	0.543	
Paternal Education (Highest Level) = A level	0.069	0.106	0.098	0.515	
Paternal Education (Highest Level) = Degree	0.124	0.129	0.101	0.337	
Sex = Female	0.909	0.063	0.009	0.000	*

SE = standard error. RIV = relative increase in variance. The crowding index was calculated as the number of household members per room. Baseline characteristics included sex = male, parity = 0, crowding index < 0.5, maternal and paternal social classes = I, maternal marital status at enrollment = never married, and the highest level of maternal and paternal education = Certificate of Secondary Education (CSE). For parental social class: I = professional occupations, II = managerial and technical occupations, III (non-manual) = skilled non-manual occupations, III (manual) = skilled manual occupations, IV = partly skilled occupations, and V = unskilled occupations. P<0.05 = *.

© 2026 Feibel A et al. *JAMA Network Open*.

eTable 18. Regression Table Between Paternal Crown-Crisp Experiential Index (CCEI) Anxiety Subscores and Offspring Age 25 Screen for Adult Anxiety Related Disorders (SCAARED) Score

Term	Beta	SE	RIV	p value	p < 0.05
PRS Panic Disorder Offspring	0.010	0.044	0.349	0.823	
PRS Bipolar Disorder Offspring	-0.038	0.053	0.248	0.472	
PRS Major Depressive Disorder Offspring	0.220	0.054	0.357	0.000	*
PRS Schizophrenia Offspring	0.052	0.058	0.347	0.371	
PRS Alcohol Use Disorder Offspring	0.028	0.048	0.310	0.566	
PRS Anxiety Offspring	0.013	0.048	0.291	0.783	
PRS Panic Disorder Maternal	0.008	0.045	0.356	0.865	
PRS Bipolar Disorder Maternal	-0.016	0.056	0.391	0.780	
PRS Major Depressive Disorder Maternal	-0.001	0.053	0.330	0.991	
PRS Schizophrenia Maternal	0.016	0.056	0.335	0.782	
PRS Alcohol Use Disorder Maternal	-0.030	0.048	0.283	0.527	
PRS Anxiety Maternal	0.012	0.049	0.355	0.812	
Parity = 1	-0.040	0.087	0.043	0.644	
Parity >= 2	0.054	0.121	0.051	0.655	
Crowding Index >0.5 - 0.75	0.144	0.090	0.055	0.109	
Crowding Index >0.75 - 1	0.260	0.124	0.059	0.035	*
Crowding Index > 1	-0.046	0.212	0.047	0.829	
Maternal Social Class = II	0.076	0.142	0.115	0.594	
Maternal Social Class = III (non-manual)	0.063	0.155	0.127	0.684	
Maternal Social Class = III (manual)	0.234	0.213	0.126	0.272	
Maternal Social Class = IV	0.061	0.208	0.172	0.769	
Maternal Social Class = V	0.048	0.375	0.034	0.899	
Paternal Social Class = II	0.101	0.106	0.061	0.341	
Paternal Social Class = III (non-manual)	-0.017	0.138	0.069	0.903	
Paternal Social Class = III (manual)	0.051	0.130	0.104	0.698	
Paternal Social Class = IV	0.067	0.174	0.074	0.701	
Paternal Social Class = V	-0.026	0.306	0.023	0.933	
Maternal Marital Status at Enrollment = 1st Marriage	-0.082	0.118	0.038	0.486	
Maternal Marital Status at Enrollment = 2nd or 3rd Marriage	-0.113	0.175	0.032	0.519	
Maternal Marital Status at Enrollment = Widowed or Divorced or Separated	-0.003	0.200	0.029	0.987	

© 2026 Feibel A et al. *JAMA Network Open*.

Term	Beta	SE	RIV	p value	p < 0.05
Maternal Age (Years)	0.016	0.011	0.021	0.151	
Paternal Age (Years)	-0.010	0.009	0.023	0.239	
Maternal Education (Highest Level) = Vocational	-0.413	0.174	0.085	0.018	*
Maternal Education (Highest Level) = O level	-0.154	0.136	0.081	0.255	
Maternal Education (Highest Level) = A level	-0.185	0.147	0.071	0.208	
Maternal Education (Highest Level) = Degree	-0.359	0.170	0.063	0.035	*
Paternal Education (Highest Level) = Vocational	0.092	0.158	0.079	0.561	
Paternal Education (Highest Level) = O level	0.087	0.126	0.079	0.490	
Paternal Education (Highest Level) = A level	0.190	0.121	0.071	0.116	
Paternal Education (Highest Level) = Degree	0.222	0.144	0.064	0.122	
Sex = Female	0.900	0.070	0.013	0.000	*

SE = standard error. RIV = relative increase in variance. The crowding index was calculated as the number of household members per room. Baseline characteristics included sex = male, parity = 0, crowding index < 0.5, maternal and paternal social classes = I, maternal marital status at enrollment = never married, and the highest level of maternal and paternal education = Certificate of Secondary Education (CSE). For parental social class: I = professional occupations, II = managerial and technical occupations, III (non-manual) = skilled non-manual occupations, III (manual) = skilled manual occupations, IV = partly skilled occupations, and V = unskilled occupations. P<0.05 = *.

© 2026 Feibel A et al. *JAMA Network Open*.

eTable 19. Regression Table Between Maternal Crown-Crisp Experiential Index (CCEI) Anxiety Subscores and Offspring "Definite" or "Suspected" Psychotic Experiences From 12-24 (Measured at 24 Using the Psychotic-Like Symptoms Interview [PLIKSi] Interview)

Term	Beta	SE	RIV	p value	p < 0.05
Intercept	-3.150	0.628	0.085	0.000	*
PRS Panic Disorder Offspring	-0.055	0.076	0.261	0.467	
PRS Bipolar Disorder Offspring	-0.036	0.094	0.274	0.698	
PRS Major Depressive Disorder Offspring	0.172	0.091	0.301	0.058	
PRS Schizophrenia Offspring	0.033	0.100	0.333	0.738	
PRS Alcohol Use Disorder Offspring	0.046	0.086	0.346	0.593	
PRS Anxiety Offspring	-0.018	0.084	0.249	0.828	
PRS Panic Disorder Maternal	0.048	0.076	0.289	0.528	
PRS Bipolar Disorder Maternal	0.021	0.096	0.317	0.829	
PRS Major Depressive Disorder Maternal	-0.017	0.092	0.278	0.850	
PRS Schizophrenia Maternal	-0.145	0.098	0.340	0.140	
PRS Alcohol Use Disorder Maternal	0.007	0.087	0.354	0.934	
PRS Anxiety Maternal	0.068	0.084	0.278	0.419	
Parity = 1	-0.070	0.154	0.083	0.647	
Parity >= 2	-0.459	0.220	0.090	0.037	*
Crowding Index >0.5 - 0.75	0.037	0.162	0.085	0.820	
Crowding Index >0.75 - 1	0.421	0.201	0.098	0.036	*
Crowding Index > 1	0.257	0.314	0.043	0.412	
Maternal Social Class = II	0.401	0.292	0.163	0.169	
Maternal Social Class = III (non-manual)	0.631	0.312	0.184	0.044	*
Maternal Social Class = III (manual)	0.601	0.389	0.137	0.122	
Maternal Social Class = IV	0.753	0.373	0.185	0.044	*
Maternal Social Class = V	1.087	0.558	0.053	0.051	
Paternal Social Class = II	0.096	0.206	0.063	0.640	
Paternal Social Class = III (non-manual)	0.003	0.266	0.112	0.991	
Paternal Social Class = III (manual)	0.151	0.238	0.087	0.527	
Paternal Social Class = IV	0.125	0.307	0.091	0.684	
Paternal Social Class = V	0.185	0.474	0.024	0.696	
Maternal Marital Status at Enrollment = 1st Marriage	-0.484	0.170	0.046	0.005	*
Maternal Marital Status at Enrollment = 2nd or 3rd Marriage	-0.187	0.278	0.056	0.501	

© 2026 Feibel A et al. *JAMA Network Open*.

Term	Beta	SE	RIV	p value	p < 0.05
Maternal Marital Status at Enrollment = Widowed or Divorced or Separated	-0.040	0.285	0.045	0.887	
Maternal Age (Years)	-0.007	0.019	0.032	0.692	
Paternal Age (Years)	0.022	0.013	0.045	0.099	
Maternal Education (Highest Level) = Vocational	0.255	0.266	0.053	0.337	
Maternal Education (Highest Level) = O level	0.114	0.218	0.055	0.601	
Maternal Education (Highest Level) = A level	-0.249	0.248	0.047	0.316	
Maternal Education (Highest Level) = Degree	0.199	0.286	0.042	0.486	
Paternal Education (Highest Level) = Vocational	0.124	0.258	0.088	0.630	
Paternal Education (Highest Level) = O level	-0.105	0.210	0.120	0.619	
Paternal Education (Highest Level) = A level	0.024	0.201	0.123	0.906	
Paternal Education (Highest Level) = Degree	0.218	0.248	0.093	0.381	
Sex = Female	-0.176	0.119	0.005	0.139	

SE = standard error. RIV = relative increase in variance. The crowding index was calculated as the number of household members per room. Baseline characteristics included sex = male, parity = 0, crowding index < 0.5, maternal and paternal social classes = I, maternal marital status at enrollment = never married, and the highest level of maternal and paternal education = Certificate of Secondary Education (CSE). For parental social class: I = professional occupations, II = managerial and technical occupations, III (non-manual) = skilled non-manual occupations, III (manual) = skilled manual occupations, IV = partly skilled occupations, and V = unskilled occupations. P<0.05 = *.

© 2026 Feibel A et al. *JAMA Network Open*.

eTable 20. Regression Table Between Paternal Crown-Crisp Experiential Index (CCEI) Anxiety Subscores and Offspring "Definite" or "Suspected" Psychotic Experiences From 12-24 (Measured at 24 Using the Psychotic-Like Symptoms Interview [PLIKSi] Interview)

Term	Beta	SE	RIV	p value	p < 0.05
Intercept	-2.727	0.701	0.074	0.000	*
PRS Panic Disorder Offspring	-0.104	0.085	0.266	0.223	
PRS Bipolar Disorder Offspring	-0.078	0.105	0.262	0.457	
PRS Major Depressive Disorder Offspring	0.181	0.103	0.302	0.080	
PRS Schizophrenia Offspring	0.127	0.111	0.311	0.253	
PRS Alcohol Use Disorder Offspring	0.012	0.095	0.304	0.896	
PRS Anxiety Offspring	-0.007	0.095	0.265	0.943	
PRS Panic Disorder Maternal	0.082	0.085	0.293	0.336	
PRS Bipolar Disorder Maternal	0.003	0.106	0.272	0.976	
PRS Major Depressive Disorder Maternal	0.004	0.101	0.245	0.966	
PRS Schizophrenia Maternal	-0.163	0.109	0.318	0.135	
PRS Alcohol Use Disorder Maternal	0.030	0.097	0.361	0.755	
PRS Anxiety Maternal	0.067	0.093	0.238	0.470	
Parity = 1	-0.167	0.173	0.071	0.335	
Parity >= 2	-0.392	0.245	0.081	0.109	
Crowding Index >0.5 - 0.75	0.086	0.180	0.074	0.631	
Crowding Index >0.75 - 1	0.454	0.228	0.088	0.047	*
Crowding Index > 1	0.290	0.400	0.042	0.469	
Maternal Social Class = II	0.281	0.314	0.160	0.372	
Maternal Social Class = III (non-manual)	0.636	0.340	0.193	0.061	
Maternal Social Class = III (manual)	0.637	0.426	0.152	0.135	
Maternal Social Class = IV	0.591	0.420	0.186	0.160	
Maternal Social Class = V	0.041	0.826	0.034	0.961	
Paternal Social Class = II	0.240	0.222	0.052	0.280	
Paternal Social Class = III (non-manual)	0.117	0.287	0.064	0.684	
Paternal Social Class = III (manual)	0.296	0.261	0.072	0.258	
Paternal Social Class = IV	-0.025	0.359	0.061	0.944	
Paternal Social Class = V	0.334	0.554	0.019	0.547	
Maternal Marital Status at Enrollment = 1st Marriage	-0.238	0.211	0.050	0.259	
Maternal Marital Status at Enrollment = 2nd or 3rd Marriage	0.056	0.318	0.049	0.861	

© 2026 Feibel A et al. *JAMA Network Open*.

Term	Beta	SE	RIV	p value	p < 0.05
Maternal Marital Status at Enrollment = Widowed or Divorced or Separated	0.134	0.346	0.044	0.699	
Maternal Age (Years)	-0.014	0.021	0.031	0.518	
Paternal Age (Years)	0.019	0.016	0.022	0.223	
Maternal Education (Highest Level) = Vocational	0.162	0.305	0.059	0.596	
Maternal Education (Highest Level) = O level	-0.113	0.255	0.068	0.657	
Maternal Education (Highest Level) = A level	-0.464	0.290	0.068	0.109	
Maternal Education (Highest Level) = Degree	-0.053	0.328	0.062	0.873	
Paternal Education (Highest Level) = Vocational	-0.056	0.305	0.068	0.854	
Paternal Education (Highest Level) = O level	-0.171	0.240	0.100	0.477	
Paternal Education (Highest Level) = A level	-0.004	0.228	0.079	0.987	
Paternal Education (Highest Level) = Degree	0.297	0.276	0.070	0.282	
Sex = Female	-0.273	0.132	0.006	0.039	*

SE = standard error. RIV = relative increase in variance. The crowding index was calculated as the number of household members per room. Baseline characteristics included sex = male, parity = 0, crowding index < 0.5, maternal and paternal social classes = I, maternal marital status at enrollment = never married, and the highest level of maternal and paternal education = Certificate of Secondary Education (CSE). For parental social class: I = professional occupations, II = managerial and technical occupations, III (non-manual) = skilled non-manual occupations, III (manual) = skilled manual occupations, IV = partly skilled occupations, and V = unskilled occupations. P<0.05 = *.

© 2026 Feibel A et al. *JAMA Network Open*.

eTable 21. Regression Table Between Maternal Crown-Crisp Experiential Index (CCEI) Anxiety Subscores and Adult Offspring Alcohol Use Disorder Identification Test (AUDIT) Score

Term	Beta	SE	RIV	p value	p < 0.05
PRS Panic Disorder Offspring	-0.004	0.040	0.304	0.910	
PRS Bipolar Disorder Offspring	-0.017	0.050	0.348	0.741	
PRS Major Depressive Disorder Offspring	-0.052	0.050	0.403	0.297	
PRS Schizophrenia Offspring	0.028	0.053	0.391	0.597	
PRS Alcohol Use Disorder Offspring	0.138	0.044	0.338	0.002	*
PRS Anxiety Offspring	-0.046	0.045	0.346	0.307	
PRS Panic Disorder Maternal	0.024	0.041	0.373	0.565	
PRS Bipolar Disorder Maternal	-0.008	0.051	0.337	0.871	
PRS Major Depressive Disorder Maternal	0.003	0.049	0.392	0.955	
PRS Schizophrenia Maternal	0.012	0.053	0.366	0.816	
PRS Alcohol Use Disorder Maternal	0.029	0.044	0.315	0.517	
PRS Anxiety Maternal	-0.029	0.045	0.334	0.522	
Parity = 1	0.167	0.079	0.038	0.034	*
Parity >= 2	0.229	0.113	0.062	0.043	*
Crowding Index >0.5 - 0.75	-0.137	0.082	0.053	0.094	
Crowding Index >0.75 - 1	-0.323	0.114	0.084	0.005	*
Crowding Index > 1	-0.465	0.195	0.066	0.017	*
Maternal Social Class = II	-0.055	0.130	0.142	0.672	
Maternal Social Class = III (non-manual)	-0.107	0.141	0.146	0.450	
Maternal Social Class = III (manual)	-0.059	0.189	0.115	0.754	
Maternal Social Class = IV	-0.110	0.186	0.168	0.552	
Maternal Social Class = V	-0.718	0.352	0.036	0.041	*
Paternal Social Class = II	0.093	0.098	0.050	0.347	
Paternal Social Class = III (non-manual)	0.008	0.127	0.077	0.947	
Paternal Social Class = III (manual)	-0.007	0.118	0.069	0.951	
Paternal Social Class = IV	0.110	0.156	0.072	0.478	
Paternal Social Class = V	0.364	0.269	0.015	0.177	
Maternal Marital Status at Enrollment = 1st Marriage	-0.357	0.105	0.038	0.001	*
Maternal Marital Status at Enrollment = 2nd or 3rd Marriage	-0.623	0.161	0.047	0.000	*
Maternal Marital Status at Enrollment = Widowed or Divorced or Separated	-0.375	0.179	0.040	0.036	*

© 2026 Feibel A et al. *JAMA Network Open*.

Term	Beta	SE	RIV	p value	p < 0.05
Maternal Age (Years)	0.023	0.010	0.030	0.022	*
Paternal Age (Years)	-0.009	0.008	0.034	0.241	
Maternal Education (Highest Level) = Vocational	0.061	0.157	0.057	0.698	
Maternal Education (Highest Level) = O level	0.245	0.122	0.059	0.044	*
Maternal Education (Highest Level) = A level	0.312	0.132	0.070	0.019	*
Maternal Education (Highest Level) = Degree	0.382	0.154	0.064	0.013	*
Paternal Education (Highest Level) = Vocational	-0.048	0.150	0.069	0.747	
Paternal Education (Highest Level) = O level	-0.099	0.112	0.085	0.376	
Paternal Education (Highest Level) = A level	0.058	0.107	0.075	0.586	
Paternal Education (Highest Level) = Degree	0.145	0.129	0.073	0.262	
Sex = Female	-0.431	0.064	0.006	0.000	*

SE = standard error. RIV = relative increase in variance. The crowding index was calculated as the number of household members per room. Baseline characteristics included sex = male, parity = 0, crowding index < 0.5, maternal and paternal social classes = I, maternal marital status at enrollment = never married, and the highest level of maternal and paternal education = Certificate of Secondary Education (CSE). For parental social class: I = professional occupations, II = managerial and technical occupations, III (non-manual) = skilled non-manual occupations, III (manual) = skilled manual occupations, IV = partly skilled occupations, and V = unskilled occupations. P<0.05 = *.

© 2026 Feibel A et al. *JAMA Network Open*.

eTable 22. Regression Table Between Paternal Crown-Crisp Experiential Index (CCEI) Anxiety Subscores and Adult Offspring Alcohol Use Disorder Identification Test (AUDIT) Score

Term	Beta	SE	RIV	p value	p < 0.05
PRS Panic Disorder Offspring	0.001	0.044	0.294	0.986	
PRS Bipolar Disorder Offspring	-0.044	0.055	0.313	0.429	
PRS Major Depressive Disorder Offspring	-0.069	0.055	0.372	0.206	
PRS Schizophrenia Offspring	0.055	0.059	0.380	0.352	
PRS Alcohol Use Disorder Offspring	0.138	0.049	0.320	0.005	*
PRS Anxiety Offspring	-0.048	0.049	0.316	0.328	
PRS Panic Disorder Maternal	0.047	0.044	0.337	0.285	
PRS Bipolar Disorder Maternal	0.012	0.056	0.316	0.824	
PRS Major Depressive Disorder Maternal	0.049	0.055	0.388	0.375	
PRS Schizophrenia Maternal	-0.005	0.059	0.370	0.934	
PRS Alcohol Use Disorder Maternal	0.050	0.049	0.329	0.313	
PRS Anxiety Maternal	-0.073	0.049	0.307	0.137	
Parity = 1	0.137	0.089	0.046	0.124	
Parity >= 2	0.226	0.127	0.068	0.076	
Crowding Index >0.5 - 0.75	-0.176	0.091	0.052	0.054	
Crowding Index >0.75 - 1	-0.441	0.129	0.084	0.001	*
Crowding Index > 1	-0.499	0.231	0.080	0.031	*
Maternal Social Class = II	-0.085	0.140	0.129	0.541	
Maternal Social Class = III (non-manual)	-0.104	0.154	0.159	0.502	
Maternal Social Class = III (manual)	-0.023	0.210	0.115	0.911	
Maternal Social Class = IV	-0.043	0.206	0.161	0.835	
Maternal Social Class = V	-0.592	0.391	0.027	0.131	
Paternal Social Class = II	0.149	0.106	0.039	0.160	
Paternal Social Class = III (non-manual)	0.051	0.137	0.052	0.712	
Paternal Social Class = III (manual)	0.069	0.128	0.044	0.591	
Paternal Social Class = IV	0.236	0.175	0.057	0.178	
Paternal Social Class = V	0.499	0.313	0.017	0.111	
Maternal Marital Status at Enrollment = 1st Marriage	-0.281	0.123	0.034	0.022	*
Maternal Marital Status at Enrollment = 2nd or 3rd Marriage	-0.390	0.182	0.038	0.032	*
Maternal Marital Status at Enrollment = Widowed or Divorced or Separated	-0.124	0.207	0.029	0.548	

© 2026 Feibel A et al. *JAMA Network Open*.

Term	Beta	SE	RIV	p value	p < 0.05
Maternal Age (Years)	0.017	0.011	0.019	0.147	
Paternal Age (Years)	-0.011	0.009	0.014	0.218	
Maternal Education (Highest Level) = Vocational	0.090	0.180	0.043	0.618	
Maternal Education (Highest Level) = O level	0.201	0.141	0.048	0.154	
Maternal Education (Highest Level) = A level	0.268	0.152	0.055	0.078	
Maternal Education (Highest Level) = Degree	0.381	0.175	0.063	0.029	*
Paternal Education (Highest Level) = Vocational	-0.063	0.170	0.056	0.710	
Paternal Education (Highest Level) = O level	-0.129	0.127	0.060	0.309	
Paternal Education (Highest Level) = A level	0.069	0.122	0.053	0.572	
Paternal Education (Highest Level) = Degree	0.180	0.144	0.057	0.211	
Sex = Female	-0.403	0.071	0.006	0.000	*

SE = standard error. RIV = relative increase in variance. The crowding index was calculated as the number of household members per room. Baseline characteristics included sex = male, parity = 0, crowding index < 0.5, maternal and paternal social classes = I, maternal marital status at enrollment = never married, and the highest level of maternal and paternal education = Certificate of Secondary Education (CSE). For parental social class: I = professional occupations, II = managerial and technical occupations, III (non-manual) = skilled non-manual occupations, III (manual) = skilled manual occupations, IV = partly skilled occupations, and V = unskilled occupations. P<0.05 = *.

© 2026 Feibel A et al. *JAMA Network Open*.

eFigure 1. Marginal Distributions and Correlations Between Adult Mental Health Outcomes

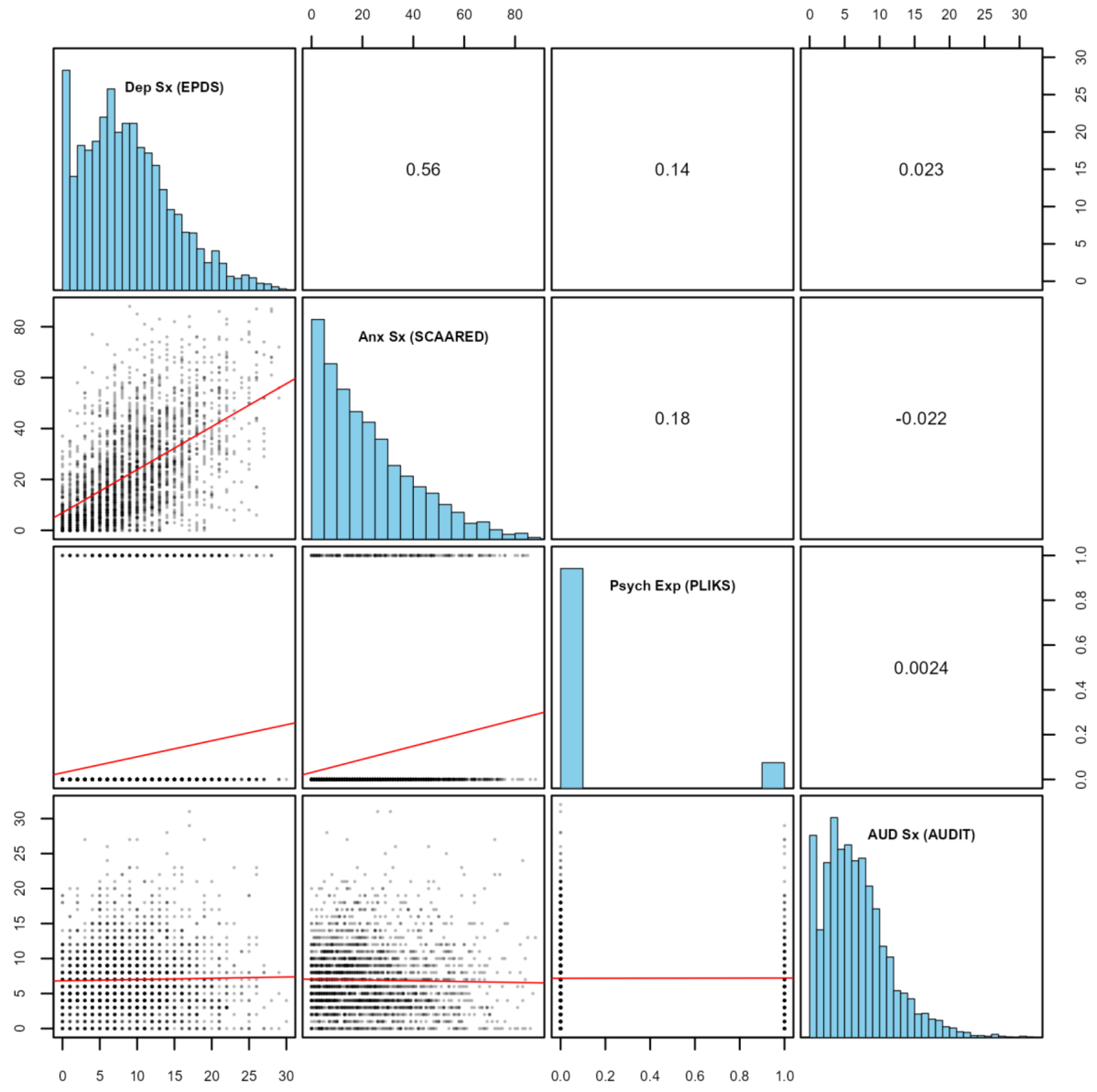

Marginal distributions are displayed as histograms along the diagonal. Pairwise Pearson's correlations are illustrated using scatterplots in the lower triangle, and the corresponding correlation values are shown in the upper triangle. The EPDS, SCAARED, and AUDIT scores are bounded non-negative integers and exhibit a positive skew and show zero inflation. We observe strong correlation between EPDS and SCAARED and a lesser correlation with PLIKS. Dep Sx = Depression Symptoms, EPDS = Edinburgh Postnatal Depression Scale, Anx Sx = Anxiety Symptoms, SCAARED = Screen for Adult Anxiety-Related Disorders, Psych Exp = Psychotic Experiences, PLIKS = Psychosis-Like Symptoms (assessed via PLIKSi = Psychosis-Like Symptoms Interview), AUD Sx = Alcohol Use Disorder Symptoms, AUDIT = Alcohol Use Disorders Identification Test.

© 2026 Feibel A et al. *JAMA Network Open*.

eFigure 2. Correlation Plots of Maternal and Paternal Edinburgh Postnatal Depression Scale (EPDS) Scores and Crown-Crisp Experiential Index (CCEI) Anxiety Subscores Across Multiple Measured Time Points From 18 Weeks' Gestation to 21 Years of Age (for EPDS) and From 18 Weeks' Gestation to 6 Years of Age (for CCEI)

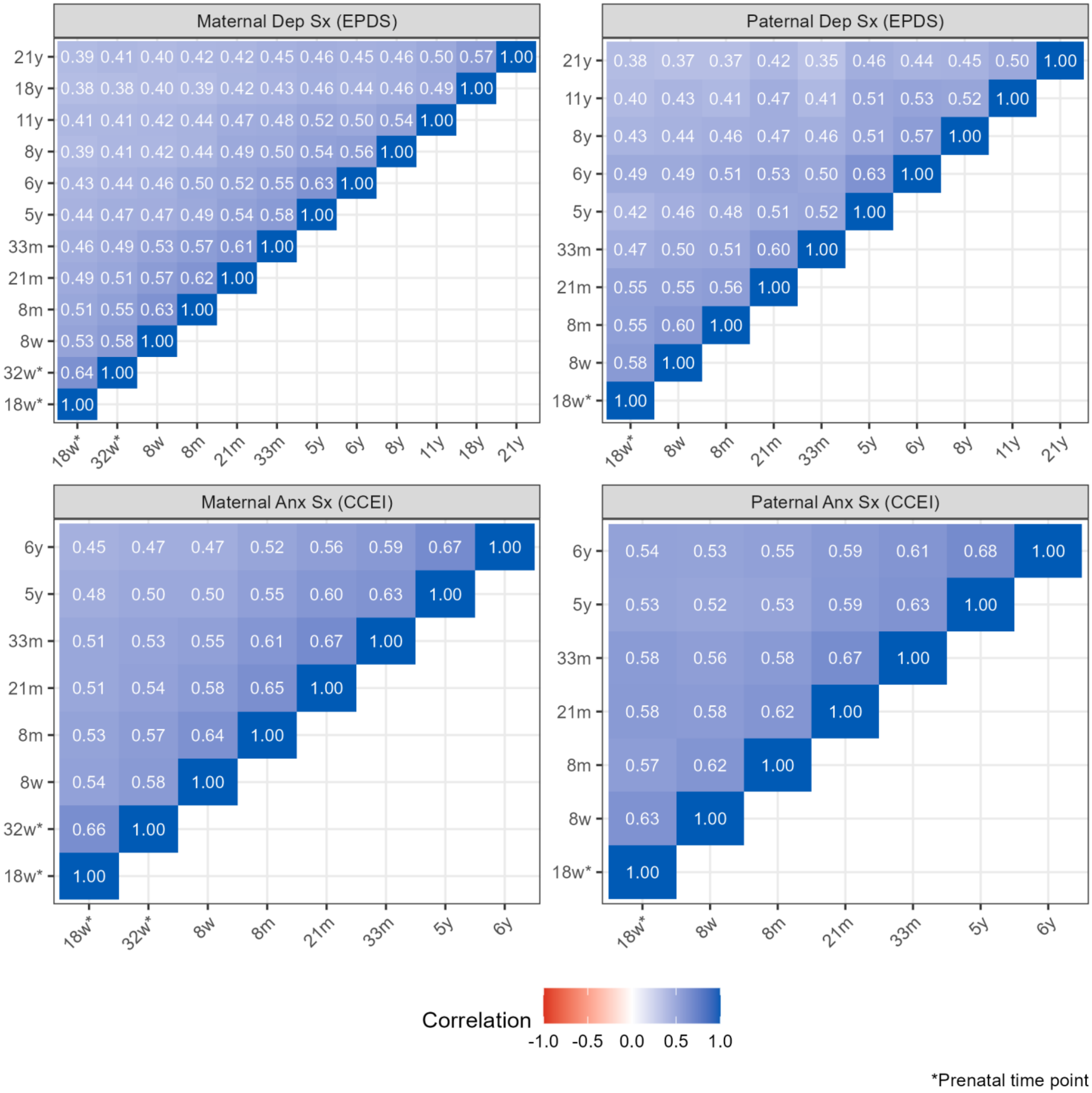

Pairwise Pearson's correlations are illustrated using heat maps with corresponding correlation values embedded. Dep Sx = Depression Symptoms, Anx Sx = Anxiety Sx.

© 2026 Feibel A et al. *JAMA Network Open*.

eFigure 3. Adjusted Odds Ratio (AOR) and Longitudinal Effect of Parental EPDS on Adult Offspring Alcohol Use Disorder Identification Test (AUDIT) Score

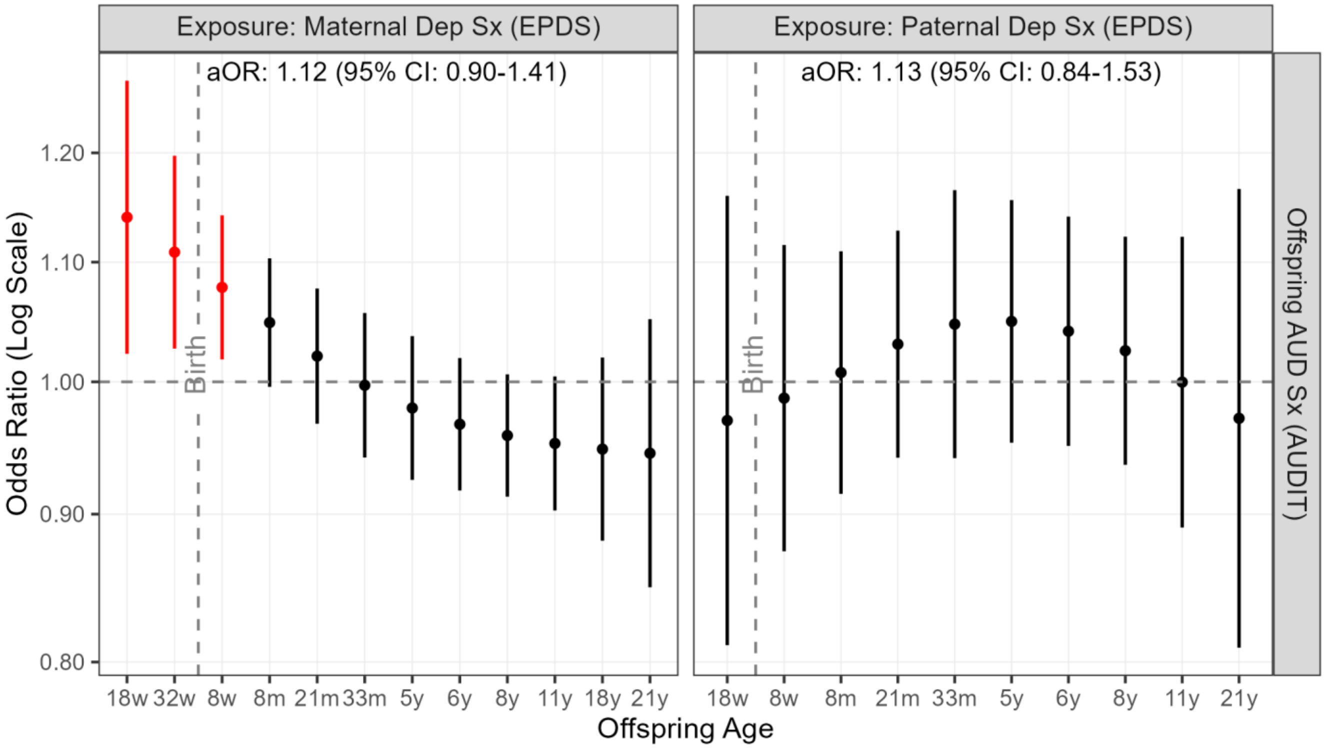

The aOR is defined as the odds of offspring having a clinically significant symptoms of mental illness after being exposed to a parental EPDS score of 13 across all measured time points (compared to no exposure to parental depression (EPDS = 0)). Measures of adult offspring mental health are represented row-wise. The color red indicates effect statistically different than null. The odds ratio is on the y-axis and offspring age on the x-axis (where 18w and 32w denote prenatal time points). The point estimates and 95% confidence intervals (CI) are the effect of raising parental scores from 0 to 13 at each measured time point on the odds of offspring having clinically significant measure of mental health. EPDS = Edinburgh Postnatal Depression Scale, CCEI = Crown-Crisp Experiential Index, SCAARED = Screen for Adult Anxiety Related Disorders, and PEs = psychotic experiences (determined in semi-structured interviews). Clinically significant symptoms of mental illness were defined as EPDS ≥ 13, SCAARED ≥ 23, and PEs being “present” or “suspected” based on interviewer rating.

© 2026 Feibel A et al. *JAMA Network Open*.

eFigure 4. Adjusted Odds Ratio (AOR) and Longitudinal Effect of Parental EPDS on Adult Offspring Measures of Mental Health, Distributed Lag Model Additionally Adjusted for Gestational Age, Birth Weight, Prenatal Smoking, and Alcohol Use

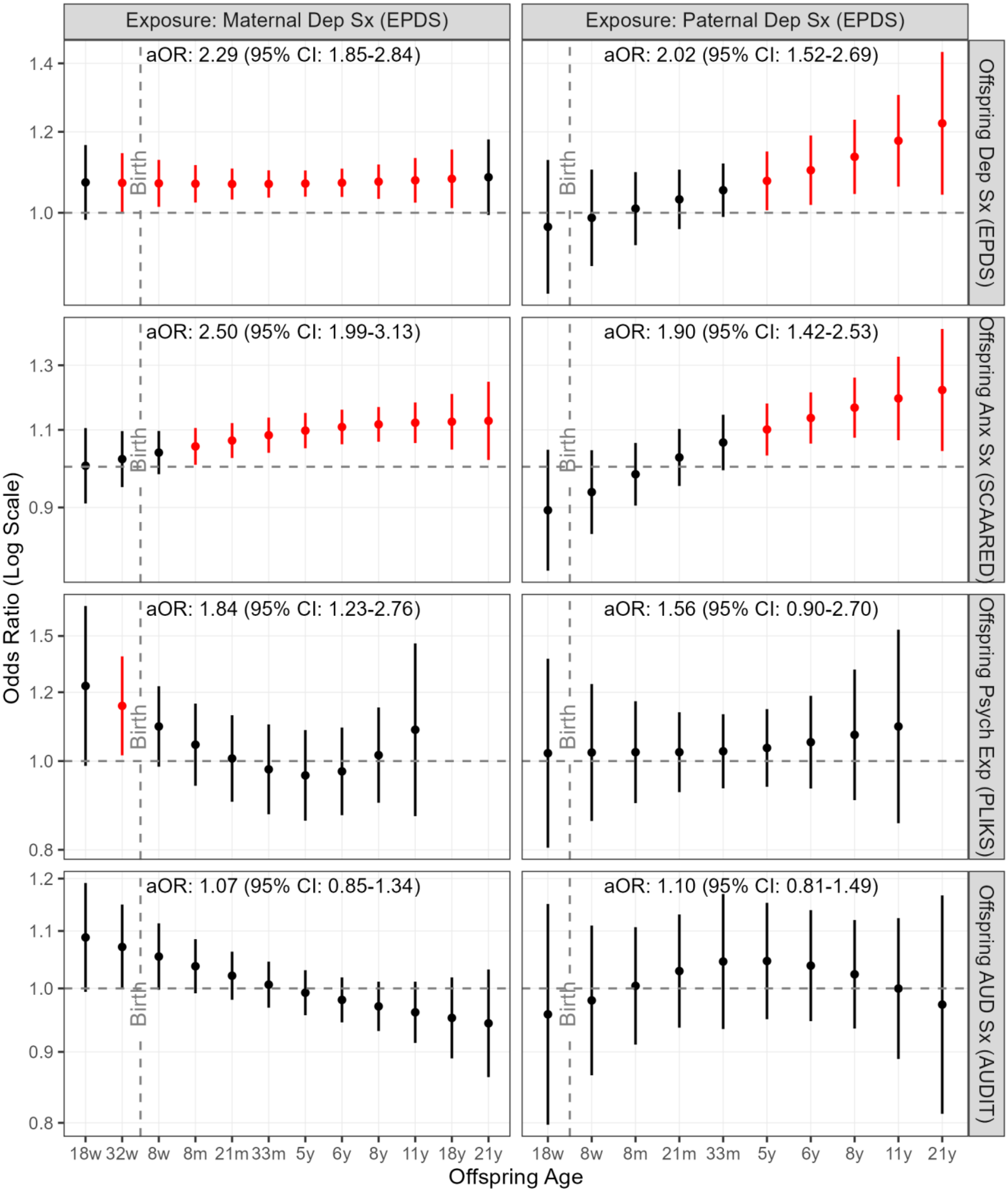

© 2026 Feibel A et al. *JAMA Network Open*.

The aOR is defined as the odds of offspring having a clinically significant symptoms of mental illness after being exposed to a parental EPDS score of 13 across all measured time points (compared to no exposure to parental depression (EPDS = 0)). Measures of adult offspring mental health are represented row-wise. The color red indicates effect statistically different than null. The odds ratio is on the y-axis and offspring age on the x-axis (where 18w and 32w denote prenatal time points). The point estimates and 95% confidence intervals (CI) are the effect of raising parental scores from 0 to 13 at each measured time point on the odds of offspring having clinically significant measure of mental health. EPDS = Edinburgh Postnatal Depression Scale, CCEI = Crown-Crisp Experiential Index, SCAARED = Screen for Adult Anxiety Related Disorders, and PEs = psychotic experiences (determined in semi-structured interviews). Clinically significant symptoms of mental illness were defined as EPDS ≥ 13, SCAARED ≥ 23, and PEs being "present" or "suspected" based on interviewer rating.

© 2026 Feibel A et al. *JAMA Network Open*.

eFigure 5. Longitudinal Effect of Parental EPDS on Adult Offspring Measures of Mental Health Using the Distributed Lag Nonlinear Models (DLNMs)

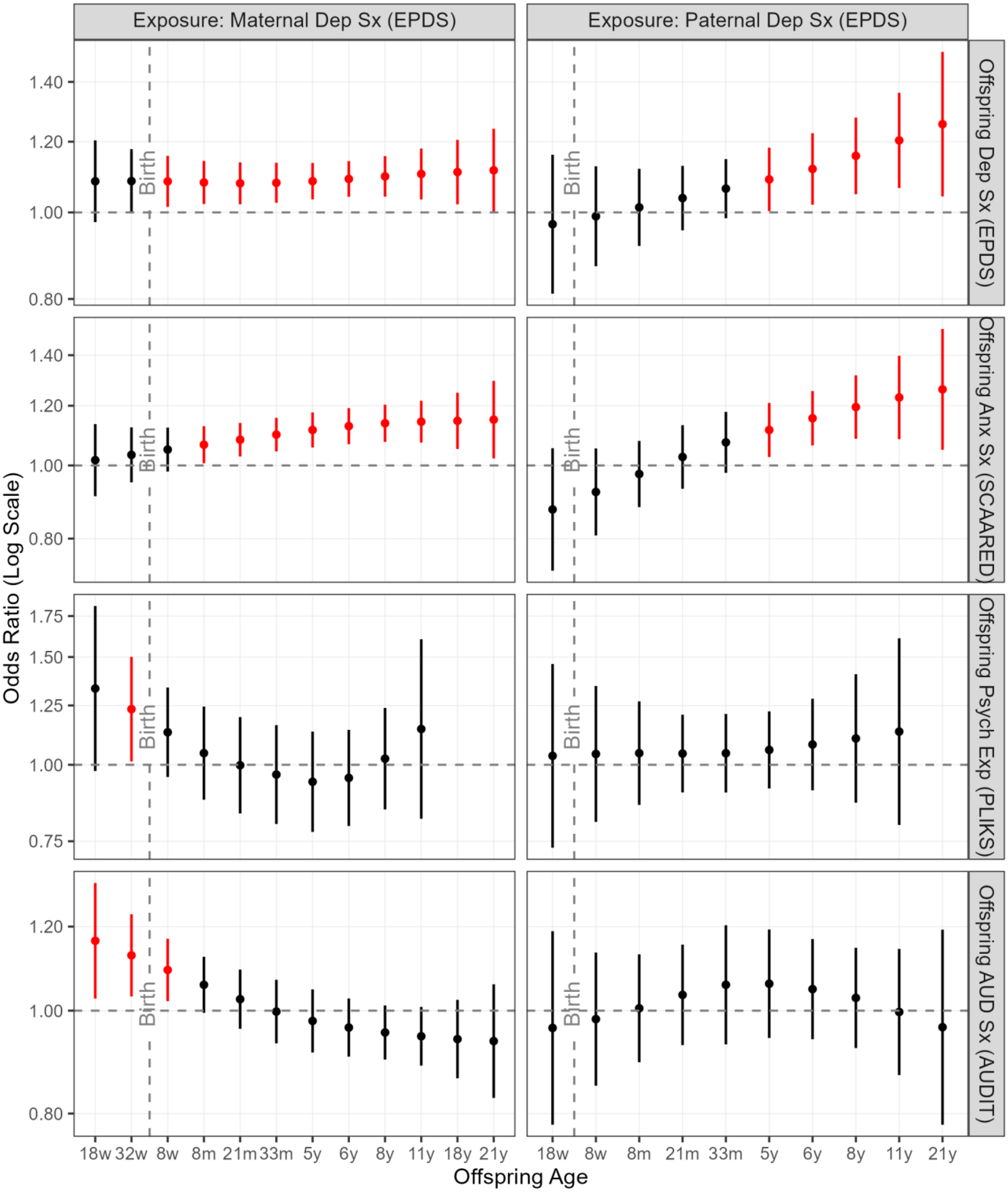

© 2026 Feibel A et al. *JAMA Network Open*.

The point estimates and 95% confidence intervals are the effect of raising parental EPDS from 0 to 15 (representative EPDS value of region with statistically significant cumulative effect across all outcomes) at each time point on the odds of offspring having a clinically significant measure of mental illness. The effects of maternal mental health are displayed in the left column, paternal in the right column. Measures of adult offspring mental health are presented row-wise. EPDS = Edinburgh Postnatal Depression Scale, SCAARED = Screen for Adult Anxiety Related Disorders, PEs = psychotic experiences (determined in semi-structured interviews), and AUDIT = Alcohol Use Disorders Identification Test. Clinically significant symptoms of mental illness were defined as EPDS ≥ 13, SCAARED ≥ 23, PEs being “present” or “suspected” based on interviewer rating, and AUDIT ≥ 8

© 2026 Feibel A et al. *JAMA Network Open*.

eFigure 6. Adjusted Odds Ratio (AOR) and Longitudinal Effect of Parental Crown-Crisp Experiential Index (CCEI) Anxiety Subscores on Adult Offspring Measures of Mental Health

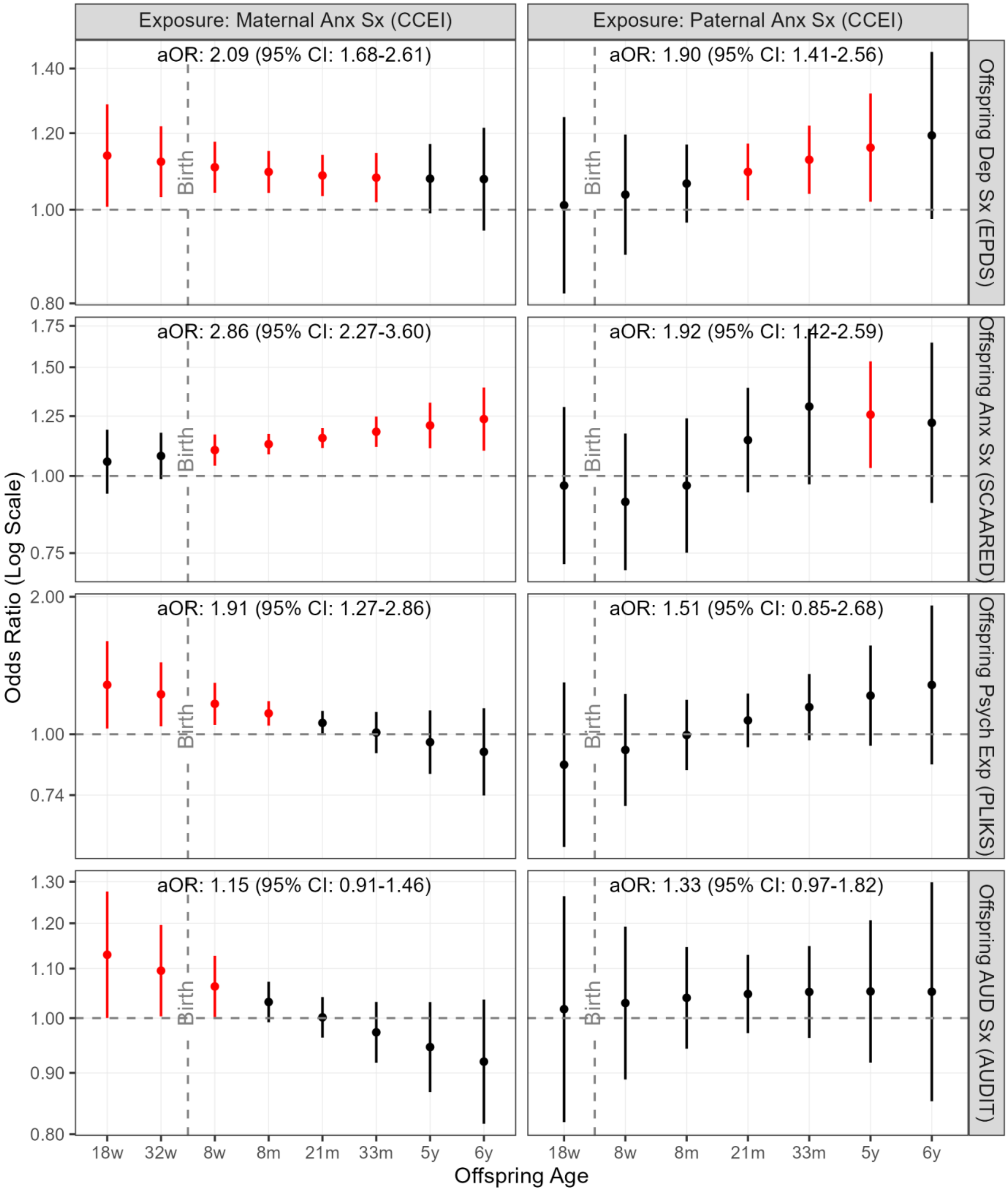

© 2026 Feibel A et al. *JAMA Network Open*.

The aOR is defined as the odds of offspring having a clinically significant symptoms of mental illness after being exposed to a parental CCEI score of 10 across all measured time points (compared to no exposure to parental anxiety (CCEI = 0)). Measures of adult offspring mental health are represented row-wise. The color red indicates effect statistically different than null. The odds ratio is on the y-axis and offspring age on the x-axis (where 18w and 32w denote prenatal time points). The point estimates and 95% confidence intervals (CI) are the effect of raising parental scores from 0 to 13 at each measured time point on the odds of offspring having clinically significant measure of mental health. EPDS = Edinburgh Postnatal Depression Scale, CCEI = Crown-Crisp Experiential Index, SCAARED = Screen for Adult Anxiety Related Disorders, and PEs = psychotic experiences (determined in semi-structured interviews). Clinically significant symptoms of mental illness were defined as EPDS ≥ 13, SCAARED ≥ 23, and PEs being "present" or "suspected" based on interviewer rating.

© 2026 Feibel A et al. *JAMA Network Open*.

eReferences

1. *1991 Census Definitions Great Britain*. (HMSO, London, 1992).
2. Josse, J. & Husson, F. missMDA: A Package for Handling Missing Values in Multivariate Data Analysis. *J. Stat. Softw.* **70**, (2016).
3. Mayer, M. *missRanger: Fast Imputation of Missing Values*. (2025).
4. Gasparrini, A., Scheipl, F., Armstrong, B. & Kenward, M. G. A Penalized Framework for Distributed Lag Non-Linear Models. *Biometrics* **73**, 938–948 (2017).
5. Demateis, D., Keller, K. P., Rojas-Rueda, D., Kioumourtzoglou, M. & Wilson, A. Penalized distributed lag interaction model: Air pollution, birth weight, and neighborhood vulnerability. *Environmetrics* **35**, e2843 (2024).

© 2026 Feibel A et al. *JAMA Network Open*.
