# Supplementary material for: Timing of Exposure to Parental Depression From Pregnancy to Young Adulthood and Mental Health in Adult Offspring
Source: JAMA Netw Open. 2026 Apr 10;9(4):e264892. doi: 10.1001/jamanetworkopen.2026.4892 (PMC13069460; doi:10.1001/jamanetworkopen.2026.4892)
Supplement: Supplement 2. — Data Sharing Statement [file jamanetwopen-e264892-s002.pdf]

# Data Sharing Statement

Feibel. Timing of Exposure to Parental Depression From Pregnancy to Young Adulthood and Mental Health in Adult Offspring. *JAMA Netw Open*. Published April 10, 2026.
doi:10.1001/jamanetworkopen.2026.4892

**Data**
**Data available:** No

**Additional Information**
**Explanation for why data not available:** All data described in this manuscript are available upon request from the ALSPAC executive in accordance with the ALSPAC data access policy see: https://www.bristol.ac.uk/alspac/researchers/access/
